# Process parameters and changes in the microbial community patterns during the first 240 days of an agricultural energy crop digester

**DOI:** 10.1186/s13568-016-0219-7

**Published:** 2016-08-02

**Authors:** Nicolas Weithmann, Alfons Rupert Weig, Ruth Freitag

**Affiliations:** 1 Process Biotechnology, Center for Energy Technology, University of Bayreuth, 95440 Bayreuth, Germany; 2 Genomics and Bioinformatics, University of Bayreuth, 95440 Bayreuth, Germany

**Keywords:** ARISA, Biogas, Community profile, Energy crop digester, Methanogenic archaea

## Abstract

Commercial biogas production takes place by complex microbial communities enclosed in controlled “technical ecosystems”. Once established, the communities tend to be resilient towards disturbances, although the relative abundance of their members may vary. The start-up phase, during which the community establishes itself, is therefore decisive for the later performance of the reactor. In this study, we followed the first 240 days of a standard agricultural energy crop digester consisting of a 400 m^3^ plug flow fermenter and a 1000 m^3^ agitated post digester, operated at 40–45 °C. The feed consisted of corn and later grass silage augmented by ground wheat. Changes in both the eubacterial and methanogenic archaeal communities were followed by automated ribosomal intergenic spacer analysis (ARISA). In addition the copy number of the methyl-coenzyme reductase A (*mcrA)*-genes found in all known methanogens were followed by quantitative PCR, while selected samples from two phases—one early, one late—of the community structure development were subjected to high throughput sequencing. Biogas volume and composition (CH_4_, CO_2_, H_2_, H_2_S, O_2_), pH, ammonia-N, and volatile fatty acids (VFA), were measured as part of the routine process control. VFA/TIC values were calculated on this basis. Whereas the total gas production of the plant established itself at about 2500 m^3^ biogas per day within the first months, the composition of the microbial communities showed distinct spatial and temporal differences over the investigated time period. Absolute values for DNA isolation procedures are difficult to certify, hence comparative results on community structures obtained using standardized ARISA with identical primers are of value. Moreover, ARISA patterns can be statistically analyzed to identify distinct subgroups and transitions between them as well as serial correlations. Thereby the microbial community and its structural development can be correlated with statistical relevance to changes in operational (feed) and process parameters (pH-value, biogas composition). In particular when augmented by deep sequencing data of judiciously chosen samples, this allows a hitherto unknown level of insight into the performance of technical biogas plants.

**Electronic supplementary material:**

The online version of this article (doi:10.1186/s13568-016-0219-7) contains supplementary material, which is available to authorized users.

## Introduction

Biogas, essentially a mixture of CO_2_ and CH_4_, is produced during the multistage anaerobic digestion of organic matter by microbial communities of eubacteria and methanogen archaea. Recently this microbial network has received considerable attention, since biogas could become an important part of a sustainable energy mix (Gallert and Winter 2002). Initially many studies in the area focused on linking productivity, i.e. biogas volume and methane content, to abiotic parameters such as feed composition, physico-chemical characteristics of the reactor environment, or aspects of process/reactor design (Simeonov et al. 1996; Angelidaki and Ahring 1992; Denac et al. 1988; Haag et al. 2003; Hill and Barth 1977; Kiely et al. 1997; Simeonov et al. 1996). More recently, advanced methods for sequencing and community profile monitoring have come into use to include also the complex microbiota responsible for biogas production in such studies. Whereas high-throughput sequencing technologies (Schlüter et al. 2008; Kröber et al. 2009; Li et al. 2013; Stolze et al. 2015; Zakrzewski et al. 2012; Campanaro et al. 2016) allow identifying the organisms composing the microbial communities at a given moment, fingerprinting-techniques like denaturing gradient gel electrophoresis (DGGE), (Bengelsdorf et al. 2013; Fliegerová et al. 2012), terminal restriction fragment length polymorphism (tRFLP), (Feng et al. 2010; Klang et al. 2015), or automated ribosomal intergenic spacer analysis (ARISA) can be used to follow and quantify changes in the microbial community profiles over time.

The latter methods in particular can be used to study the dynamic response of the microbial community to changing feed composition and environmental parameters, but also to the built up of intermediates and toxic metabolites (Rastogi et al. 2008; Mestrot et al. 2013; Siegert and Banks 2005; Hori et al. 2006; Ziganshin et al. 2013). Ideally these data can then form the basis for the design of more robust biogas production processes. However, most of the above-mentioned studies have used laboratory scale reactors to mimic technical plants (Siegert and Banks 2005; Hori et al. 2006; Ziganshin et al. 2013; Mestrot et al. 2013). The reactions of the microbial communities and their performance to changes in the process parameters was often more pronounced in such laboratory experiments than those seen in actual technical plants. It has been suggested that the small reactors present a much less diverse and in consequence less stable “technical ecosystem” than the large-scale technical units. Data from operating technical plants therefore have their particular intrinsic value.

Weiss et al. (2008, 2009) observed that the established microbiota of a municipal waste digester (80,000 t/a) composed of two reactors operated in parallel, was extremely resilient. While relative abundances may have varied in response to feed composition or process disturbances, the underlying community structure did not. Moreover, the two biogas reactors of the investigated plant had distinctly different community structures. Lucas et al. (2015), on the other hand, showed that the communities of three established energy crop digesters had similar community profiles in spite of some “historical” differences, while different community structures were found in other reactors included in their study. Lucas et al. attributed this to the fact that the three biogas reactors in question were technically identical and therefore created a similar environment. However, they were apparently also inoculated at the same time with aliquots from the same inoculum. It is therefore possible that the microbial communities and in particular those of the methanogenic archaea in biogas reactors are founder determined, i.e. settled during the start-up phase. Permanent changes of the once established community at later stages may be difficult to achieve. In this case, the start-up phase is of utmost importance for the later performance of the reactor.

Since biogas plants typically are operated continuously, opportunities to follow an initial start-up phase are rare. Some information is available on the start-up phase of sewage sludge digesters (De la Rubia et al. 2013; Liu et al. 2002; McMahon et al. 2004). Kobayashi et al. (2009) found a correlation between the quality of the inoculum and the subsequent reactor stability, while Bolzonella et al. (2003) investigated ways to shorten the start-up phase of communal waste digesters. Others focused on digesters using liquid manure (Brambilla et al. 2012; Chachkhiani et al. 2004) or industrial- and municipal-waste (Collins et al. 2003; Griffin et al. 1998; Ike et al. 2010; Williams et al. 2013; Qu et al. 2009).

Little has been published up to now on the start-up phase of agricultural energy crop digesters in spite of their exponential increase in number over the last decade. Productivity/efficiency of the biogas production is much more important in such reactors, which are exclusively focusing on energy production rather than on waste treatment. At the laboratory scale, Klang et al. (2015) presented a direct comparison between corn and sugar beet silage as feed. Both the bacterial and the archaeal communities were followed for 337 days using tRFLP followed by cloning and sequencing of the relevant bands. The results show significant differences in the established communities as a function of the feed. Disturbances like increased ammonia concentrations or low pH-values did not necessarily cause a complete breakdown of the process. Often the population was merely found to shift towards less sensitive microorganisms.

In this contribution we want to extend the database by presenting results on changes in the community profiles observed during the first 240 days of operation of a typical two stage (fermenter–post digester) agricultural energy crop digester. Bacterial and archaeal communities were followed by ARISA. Community structure data were correlated with qPCR quantification of the methyl-coenzyme reductase A (*mcrA)*-genes and data from high throughput sequencing of selected samples as well as with the biogas production and the physico-chemical process parameters.

## Materials and methods

### Biogas plant

The investigated plant was a standard EUCO Titan 185 AIO (Schmack Biogas GmbH, Schwandorf, Germany) consisting of a 30 m^2^ Pasco 20 CR unit for feeding, a 400 m^3^ plug-flow fermenter EUCO 400 TS with spool agitators and a 1000 m^3^ agitated post digester COCCUS located in Bayreuth, Germany. Fermenter and post digester are operated in sequence. Both are equipped with heating aggregates and operated between 42 and 45 °C. The plant converts ca. 3200 t of corn silage and 200 t of ground wheat per year together with a varying amount of grass silage and produces about 2600 m^3^ of biogas per day (average CH_4_ content: 55 %). Depending on the substrate composition, average residence times over both reactors compartments vary between 110 and 125 days. Gas volume/composition (CH_4_, CO_2_, H_2_, H_2_S) and temperature are recorded on a daily basis by the BIOWATCH Biogas Plant Management System (Schmack Biogas GmbH, Schwandorf, Germany). Whereas most parameters including biogas composition are determined individually for fermenter and post digester, the total biogas volume is only determined as bulk parameter.

### Start-up of the plant

To start the plant, repository content was mixed with water in the post-digester. This mixture was pumped back and forth between fermenter and post-digester several times during the next 10 days. On day 14, the fermenter was inoculated with slurry from existing biogas plants. This point in time was set as start and the first analysis was performed (sample “0”). Five days later, the first substrate (corn silage) was fed into the fermenter, this is “day 1” in the subsequent analysis. In addition, 160 kg of ionic Fe for desulfurization and 200 kg of urea (N-source) were added. Subsequently, approximately 40 kg Fe and 50 kg urea were added per week. For the first 100 days, the plant was fed exclusively with corn silage at ca. 8–11 t/d. Afterwards, grass silage was added to the feed (1–7 t/d). Starting on day 160, the feed was further augmented by adding ground wheat (0.4–1 t/d).

### Sampling

500 mL samples were collected on a weekly basis from both the fermenter and the post-digester. For biological analyses 10 mL aliquots were frozen at site and transported on dry ice together with the rest of the samples directly to the institute. Samples intended for DNA extraction were stored at −20 °C. Physico-chemical parameters were analyzed using aliquots from the unfrozen samples. Dry substance was determined by drying 5 g of the samples at 105 °C in a drying cabinet until mass constancy was reached.

### Analysis of physico-chemical parameters

pH-values and conductivities of the thoroughly vortexed samples were determined at room temperature by standard electrodes (pH10-Pen, Qcond 2400, both VWR International, Darmstadt, Germany). VFA/TIC-values (ratio of volatile fatty acids to total inorganic carbonate) were determined in gram VFA per gram of CaCO_3_ after the samples had been centrifuged at 1000×*g* for 5 min and passed through a fluted filter as proposed by Thrän et al. (2012). In addition, trace elements (DIN ISO 11885), nutrients (DIN ISO 11885), fatty acids (DIN 38409 H21), dry substance (DIN EN 12880), organic dry substance (DIN EN 12879), total nitrogen (DIN ISO 11261), and ammonia nitrogen (DIN 38406-E 5) were determined by an external service laboratory (Schmack Biogas GmbH, Schwandorf, Germany).

### Extraction of nucleic acids

For each sample, two reference specimen were subjected to DNA extraction using a slightly modified version of the improved DNA/RNA phenol extraction protocol by Griffiths et al. (2000) as proposed by Töwe et al. (2011). Briefly, samples were allowed to warm to room temperature and mixed thoroughly, followed by breakup of the cells by a 3 min vortex at 3200 rpm (Vortex Genie 2 labshaker, Scientific Industries, New York, USA) in NucleoSpin Bead Tubes (Macherey–Nagel, Düren, Germany). The obtained pellet was taken up in 150 µL of nuclease free water (AppliChem, Darmstadt, Germany). 20 µL aliquots were stored at 4 °C until analysis.

### Quantitative PCR (qPCR)

The abundance of the *mcrA*-genes in the microbial communities was quantified by real-time qPCR using an Mx3005P (Agilent, Santa Clara, USA). DNA concentrations in the cell extracts were measured by NanoDrop 2000 UV–Vis Spectrometer (Thermo Scientific, Waltham, USA) followed by individual dilution to reach a final concentration of approximately 5 ng/approach. Diluted samples were mixed with 5 µL of SybrGreen Mastermix (Kapa, Wilmington, USA), 0.2 µL ROX Low reference Dye (Kapa, Wilmington, USA) and 0.25 µL of the two primers (Eurofins, Ebersberg, Germany), namely mlas and mcrA-rev (Steinberg and Regan 2008) to give a final total volume of 10 µL. The mixture was pipetted into a low profile 96 well plate (Thermo Scientific, Erlangen, Germany), sealed with Ultra Clear Cap Strips (Thermo Scientific, Erlangen, Germany), and centrifuged for 5 min at 500×*g*. qPCR conditions were as follows: 3 min at 95 °C, then 40 cycles of denaturation at 95 °C for 30 s, annealing at 62 °C for 45 s and extension at 72 °C for 30 s. The final cycle was 95 °C for 15 s, 55 °C for 30 s and 95 °C for 30 s. For quantification a plasmid standard was prepared by cloning and ligation of a *mcrA* sequence by CloneJET PCR Cloning Kit (Thermo Scientific, Erlangen, Germany). Correctness of the DNA insert was verified by sequencing (Microsynth AG, Balgach, Switzerland).

### Automated ribosomal intergenic spacer analysis (ARISA)

For ARISA of bacterial and archaeal communities, the original protocol by Fisher and Triplett (1999) was applied in modified form as suggested by Weig et al. (2013), using 10 ng of DNA in a 12.5 µL PCR volume. Ribosomal intergenic fragments were amplified from eubacteria using primers ITSF and ITSReub (Cardinale et al. 2004) and from methanogenic archaea using primers 16S-RIS-M and 23S-RIS-M (Ciesielski et al. 2013). All primers were from biomers.net GmbH, Ulm, Germany. The forward primers were labeled with fluorescent dyes BMN-6 (ITSF) and BMN-5 (16S-RIS-M), respectively, to allow parallel detection of eubacterial and archaeal DNA fragments by capillary electrophoresis. For analysis, PCR amplification products were mixed with the MapMarker size standard (50–1200 bp, Bioventures Inc., Murfreesboro, TN, USA) and separated by capillary electrophoresis (GenomeLab GeXP Genetic Analysis System; AB Sciex Germany GmbH, Darmstadt, Germany) using an optimized protocol for long DNA fragments as recommended by the manufacturer. Electropherograms were analyzed using the Genemarker v1.95 software (SoftGenetics, State College, PA, USA). Eubacterial and archaeal fragments were scored and binned from 180 to 893 and from 530 to 893 bp, respectively, and the resulting peak intensity matrix was used for statistical analyses.

### Statistical analysis of ARISA signatures

Procedures and tools used for the statistical analysis were all embedded in the Primer v7.0.8 and Permanova + add-on v1.0.5 software (both from PRIMER-E Ltd., Lutton, United Kingdom). Raw intensity data were first normalized by square-root transformation and resemblance matrices were calculated using the Bray–Curtis similarity coefficient. Principal coordinate analyses (PCO) were conducted separately for methanogenic archaea and for eubacteria. From three-dimensional PCO scatter plots, four subgroups of data points (0–14, 23–58, 73–137, 164–233 sampling days after inoculation) could be detected, and the significant assignment of these four groups as well as between other groups (e.g. eubacteria vs. archaea, fermenter vs. post digester) was investigated by analyses of similarity (ANOSIM, 10,000 permutations). Seriation analyses along the investigated time line were conducted to test for the similarity of ARISA patterns between consecutive samples using the Spearman rank correlation method. ARISA fragment type accumulation plots were calculated for each subgroup of samples, i.e. archaea/eubacteria and fermenter/post digester, respectively. Shannon’s (H) and Simpson’s (1−λ′) indices were calculated as measures for fragment diversity and dominance for archaea and eubacteria in the fermenter and post digester communities, respectively. Correlation between eubacterial and archaeal ARISA resemblance matrices and environmental variables (log-transformed) were tested via the BEST tool implemented in Primer 7 (BIOENV method using Spearman rank correlation). Since not all environmental variables were measured during the first few days of the startup phase, only data points from day 14 onward were included in the analysis. Furthermore, the sum of normalized ARISA signals was calculated for each sampling day for fermenter and post digester samples, respectively, since only one single measurement of environmental parameters were taken, corresponding to two replicate ARISA samples. Some missing environmental data were extrapolated by applying the expectation maximum likelihood algorithm implemented in Primer 7 (1000 iterations, minimum value change: 10^−6^).

## 16S rDNA sequencing of selected samples

DNA samples (from two independent replicates) collected on days 30, 37 and 50 (phase 2), and days 178, 192 and 206 (phase 4) from the fermenter and post-digester were selected for high throughput sequencing of the amplified 16S rDNA fragments as described by Sundberg et al. (2013). PCR amplification of 16S rDNA fragments and sequencing was conducted by LGC Genomics GmbH (Berlin, Germany) according to the following protocol (kindly provided by LGC Genomics GmbH). The PCRs included about 5 ng of DNA extract, 15 pmol of each forward primer U341F 5′-NNNNNNNNNNCCTAYGGGRBGCASCAG and reverse primer U806R 5′-NNNNNNNNNNGGACTACNNGGGTATCTAAT in a 20 µL volume of MyTaq buffer containing 1.5 units MyTaq DNA polymerase (Bioline) and 2 µL of BioStabII PCR Enhancer (Sigma). For each sample, the forward and reverse primers had the same 10-nt barcode sequence. PCRs were carried out for 30 cycles using the following parameters: 2 min 96 °C pre-denaturation; 96 °C for 15 s, 50 °C for 30 s, 70 °C for 90 s. The DNA concentration of amplicons of interest was determined by gel electrophoresis. About 20 ng amplicon DNA of each sample were pooled for up to 48 samples carrying different barcodes. If needed PCRs showing low yields were further amplified for five cycles. The amplicon pools were purified with one volume AMPure XP beads (Agencourt) to remove primer dimers and other small mispriming products, followed by an additional purification on MinElute columns (Qiagen). About 100 ng of each purified amplicon pool DNA was used to construct Illumina libraries using the Ovation Rapid DR Multiplex System 1-96 (NuGEN). Illumina libraries were pooled and size selected by preparative gel electrophoresis. Sequencing (300 bp, paired-end modus) was done on an Illumina MiSeq using V3 Chemistry (Illumina). Sequencing and post-processing of the raw data was performed by LGC Genomics GmbH and included the following steps: demultiplexing of all libraries using Illumina’s bcl2fastq 1.8.4 software, sorting of reads by amplicon inline barcodes to distinguish independent samples, clipping of sequencing adapter remnants from all reads, amplification primer detection and clipping, combination of forward and reverse reads using BBMerge 34.48. The resulting 16S rDNA sequences were processed with Qiime (v1.9.1; using the pipelines ‘pick_open_reference_otus.py’, ‘biom summarize-table’, and ‘core_diversity_analyses.py’) (Caporaso et al. 2010; Edgar 2010). The sequence data were submitted to NCBI’s sequence reads archive (http://www.ncbi.nlm.nih.gov/sra/) under accession no. PRJNA328116.

## Results

### Performance of the biogas reactors

**Fig. 1:**
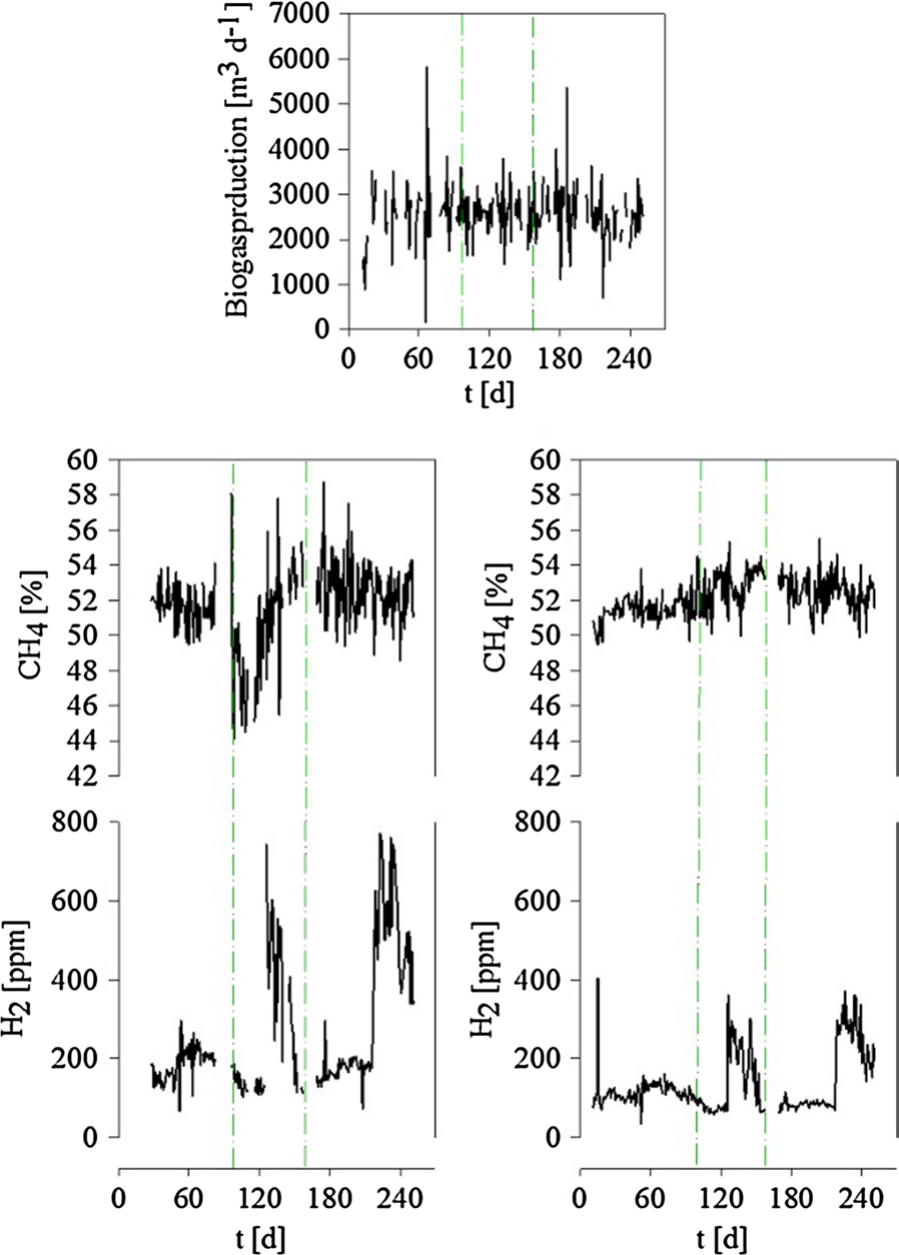
Biogas production over the observation period. *Top* Total biogas (no data available on the individual contributions of the fermenter and the post digester). *Middle* Methane content of the biogas from *left* fermenter and *right* post digester. *Bottom* H_2_-content of the biogas from *left* fermenter and *right* post digester. *Dash*-*dotted lines* mark feed changes. *First line* day 100, start of the addition of grass silage, *second line* day 160, start of the addition of ground wheat

**Fig. 2:**
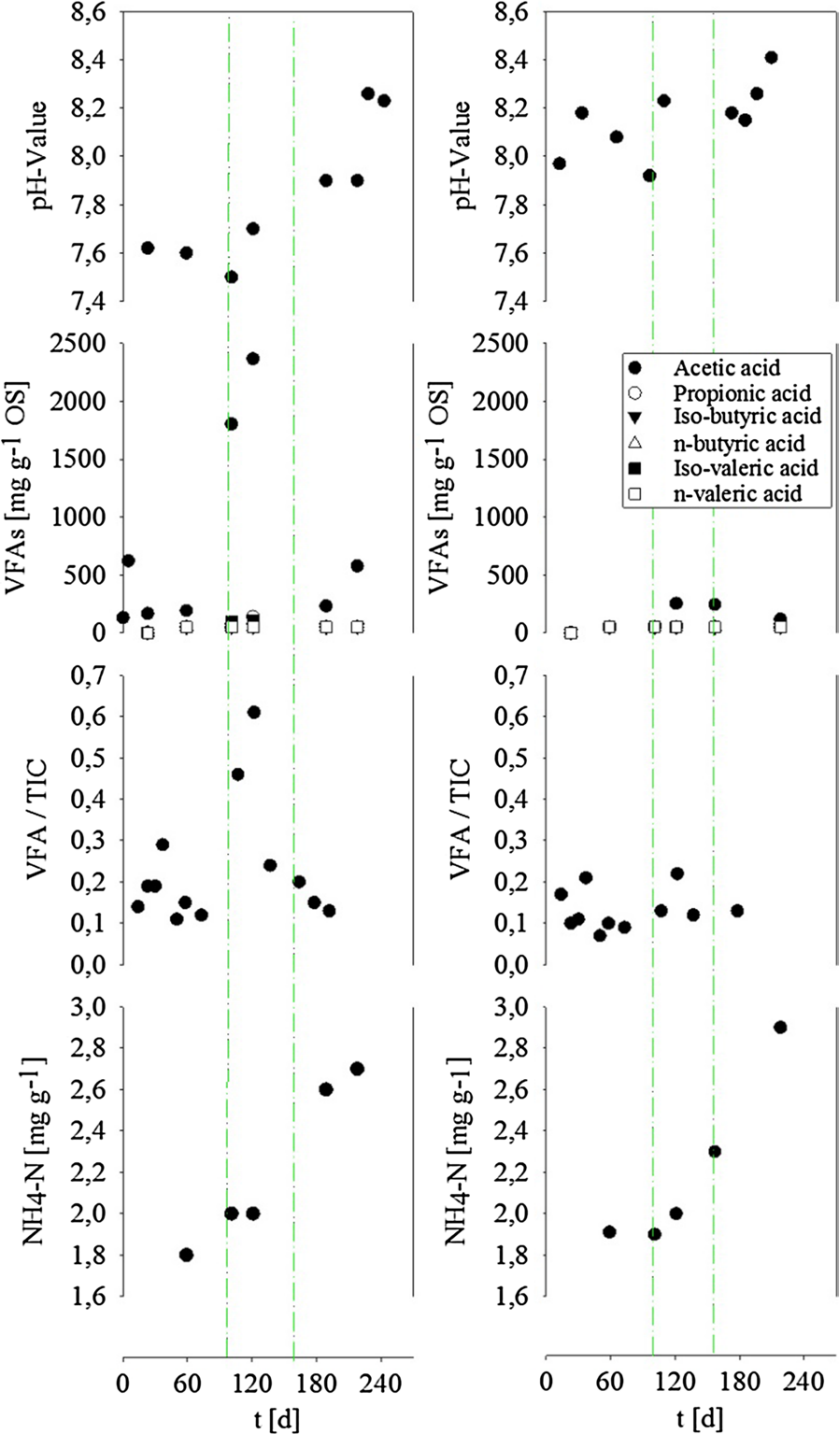
Development of abiotic process parameters over the observation period. From *top* to *bottom* pH-values, concentration of volatile fatty acids (VFA), VFA/TIC-values, ammonia-N-values in *left* fermenter and *right* post digester. *Dash*-*dotted lines* mark feed changes. *First line* day 100, start of the addition of grass silage, *second line* day 160, start of the addition of ground wheat

The two consecutive reactor compartments, fermenter and post digester, were monitored for 240 days after inoculation with sludge taken from established nearby energy crop digesters. Initially only corn silage was used as substrate. From day 100 onward grass silage was added and starting on day 160 also some ground wheat. Data on biogas production and quality are summarized in Fig. 1, while Fig. 2 summarizes changes in the pH-values and the volatile fatty acids (VFA) together with the corresponding calculated VFA/TIC-values and the ammonia-N-values. The reactors were inoculated at 36 °C and the temperature increased steadily over the next month to approximately 43 °C in the fermenter and 45 °C in the post digester. Whereas the biogas composition was analyzed separately for fermenter and post digester, the gas volume was only measured as lump value. This value stabilized early on, i.e. together with the temperature during the first month of operation.

Over the first 100 days the biogas from both reactors contained approximately 50 % methane, the H_2_ concentration was <300 ppm in the fermenter and <200 ppm in the post digester. This was followed by a 70 day-phase of more pronounced variability in the CH_4_ content, coinciding with the initiation of grass silage feeding, before the methane content finally stabilized at approximately 55 % around day 160 (coinciding with the start of the addition of ground wheat). The H_2_-content of the biogas increased in both reactor compartments during two time periods, firstly between day 120 and 150 and again after day 210 until the end of the observation period. In between, values around 200 ppm were determined. While detectable, the effect was less pronounced in the post digester. The increase in H_2_ closely followed an increase of the VFA-concentrations. The most dramatic increase was detected for the concentration of acetic acid in the fermenter, where a value of 192 mg/kg at day 59 changed to 1804 mg/kg on day 101 and up to 2367 mg/kg on days 121. Concomitantly the pH was found to decrease due to this acidification. The VFA/TIC-value, which relates the VFA content to the buffer capacity of the reactor fluids, surpassed the critical value of 0.4 in the fermenter during that time (0.6 on day 212). No concomitant decrease in pH was observed during the second phase of peaking H_2_ in either compartment. However, at that moment the ammonia-N in both fermenter and post digester had reached values close to 3 g/kg, so presumably a considerable amount of alkalinity was being produced at that time.

Safe for one isolated incident in the fermenter around day 85, oxygen levels in the biogas from both compartments were consistently below 1 %. Also around day 85 a spike in the H_2_S content of the biogas from the fermenter was observed reaching almost 900 ppm. This was not the case for the biogas from the post digester, which remained below 100 ppm H_2_S throughout. Moreover, after having spiked around day 85, the H_2_S-levels in the fermenter biogas dropped below 100 ppm on day 88 only to start increasing steadily again from day 95 onwards, reaching ca. 300 ppm at the end of the observation period.

### Analysis of the microbial communities

**Fig. 3:**
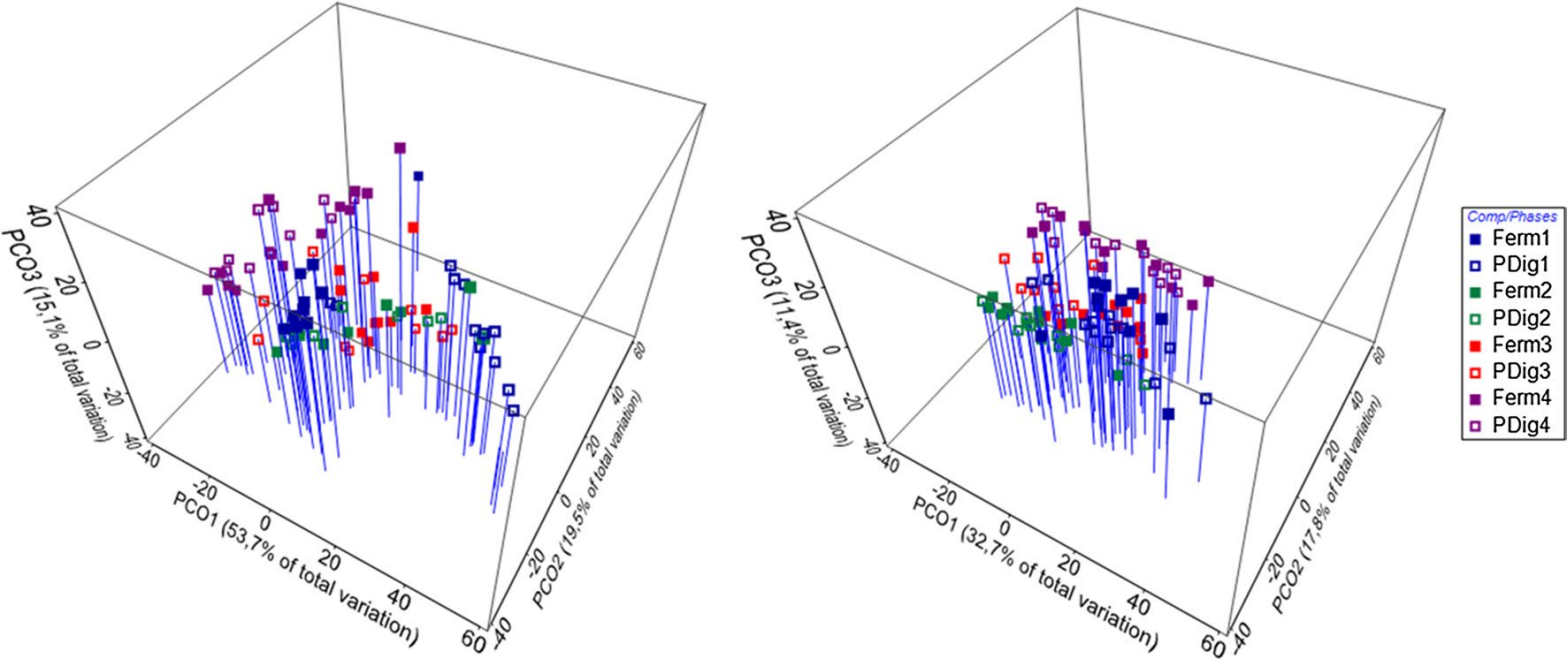
Principal coordinate analyses calculated from ARISA similarity matrices of methanogenic archaea (*left*) and eubacteria (*right*). The samples were assigned to a combination of compartments (fermenter, post digester) and time phases (I–IV): notable phase I (*blue symbols* days 0–14), phase II (*green symbols* days 23–58), phase III (*red symbols* days 73–137) and phase IV (*pink symbols* days 164–233). *Filled symbols* refer to fermenter (‘Ferm’) samples, *open symbols* to post digester (‘PDig’) samples. *Blue lines* facilitate vertical projection of the data points on the *x*, *y* plane

Principal coordinate analyses (PCO) of the ARISA resemblance matrices obtained from methanogenic archaea and eubacteria revealed that approximately 61.9 and 88.3 % of the eubacterial and archaeal ARISA signatures can be explained by three PCO axes, and four sub-clusters, which can easily be recognized (Fig. 3) and which allow the partitioning of the observation period in four distinct time phases (phase I: 0–14 days, phase II: 23–58 days, phase III: 73–137 days, phase IV: 164–233 days). In accordance the community structures differed over time for both eubacteria and methanogenic archaea. Pairwise statistical analysis (ANOSIM) comparing samples from the bacterial and archaeal sample group for a given reactor confirmed that the microbial communities differed with statistical significance for both the methanogenic archaea and the eubacteria in time phases I to IV (Table 1).

**Table 1:** Pairwise statistical comparison of similarity (ANOSIM, 10,000 permutations) for samples from the bacterial and the archaeal sample groups respectively for a given reactor (fermenter or post digester) and the indicated two time phases

Compartment	Pairs	Methanogenic archaea		Eubacteria	
		R value	p value (%)	R value	p value (%)
Fermenter	Phase I–phase II	0.373	0.02	0.709	0.01
Fermenter	Phase II–phase III	0.561	0.01	0.527	0.04
Fermenter	Phase III–phase IV	0.522	0.01	0.457	0.01
Post digester	Phase I–phase II	0.304	0.60	0.565	0.01
Post digester	Phase II–phase III	0.240	1.60	0.231	0.10
Post digester	Phase III–phase IV	0.496	0.02	0.306	0.50

Moreover, well-separated community structures were detected between fermenter and post digester during phase I (Table 2), where archaeal and bacterial communities can easily be assigned at statistically significant levels as belonging to either the fermenter or the post digester. During the subsequent phases II–IV these differences between fermenter and post digester samples disappeared and the respective archaeal and bacterial community structures start to overlap completely, save for some differences observed between the eubacterial communities in the fermenter and the post digester towards the end of phase III.

**Table 2:** Pairwise statistical comparison of similarity (ANOSIM, 10,000 permutations) for samples from the bacterial and the archaeal sample groups respectively for a given time phase and the two reactor compartments (fermenter/post digester)

	Pairs	Methanogenic archaea		Eubacteria	
		R value	p value (%)	R value	p value (%)
Phase I	Fermenter, post digester	0.596	0.01	0.463	0.01
Phase II	Fermenter, post digester	0.012	30.70	−0.029	63.40
Phase III	Fermenter, post digester	0.049	19.50	0.216	1.70
Phase IV	Fermenter, post digester	0.014	29.40	0.027	25.00

Seriation analyses on methanogenic archaea and eubacteria in the two sample groups (fermenter and post digester) showed that the ARISA fragment profiles correlate highly with the time axis in each group (Table 3).

**Table 3:** Seriation analyses (similarity of ARISA patterns between consecutive samples) within the different sample groups (fermenter/post digester, bacteria/archaea for each), using the Spearman rank correlation methods

	roh-value	p value (%)
Methanogenic archaea		
Fermenter	0.529	<0.1
Post digester	0.555	<0.1
Eubacteria		
Fermenter	0.552	<0.1
Post digester	0.498	<0.1

The corresponding rho-values were obtained using 999 permutations

Correlation between environmental factors and changes in the microbial community composition were tested by the BIOENV method available in Primer 7 software (by spearman ranking, index rho), using a maximum of five variables. Significant correlation indicated by a rho-value of 0.362 (p = 0.01) were calculated between the eubacterial ARISA pattern and a combination of gas volume per day and methane content. In case of the archaea, best correlation with a rho-value of 0.325 (p = 0.01) was found between ARISA patterns and a combination of gas volume per day, methane content, and O_2_ as variables.

**Fig. 4:**
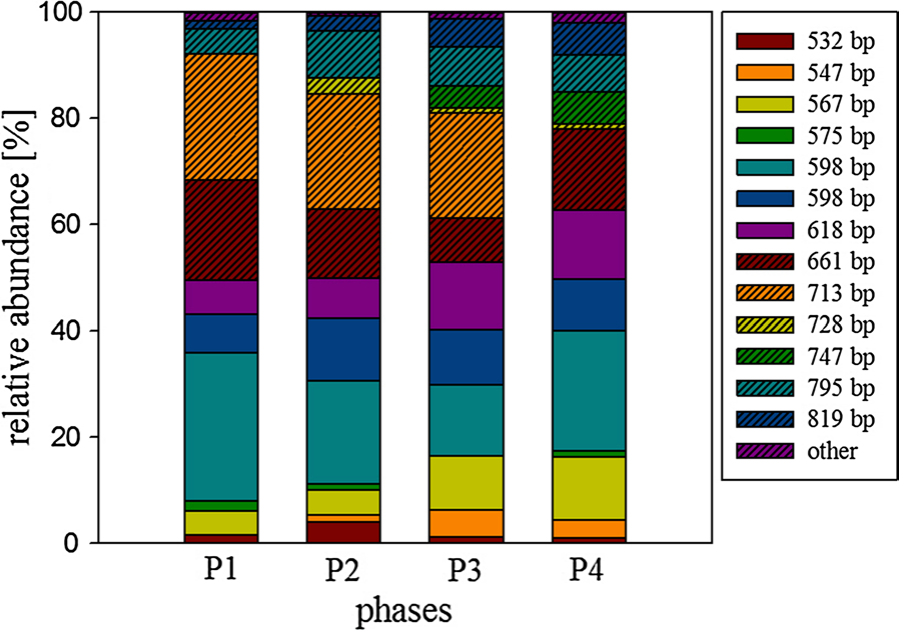
Relative abundance of the archaeal ARISA-fragments during the four time phases. Both reactor compartments were lumped in this analysis. Fragments, less than 1 % of total are summarized and displayed as “others”. Total abundance (sum of normalized values) of ARISA fragments was 251, 360, 351 and 387 for the Phases I–IV respectively

Accumulation plots based on the ARISA fragment types showed in all sample groups saturation at 16 (methanogenic archaea) and 71 (eubacteria) major fragment types (data not shown). Shannon’s index, H, as measure of biodiversity, ranged between 3.5 and 4 in case of the eubacteria, while it ranged between 1 and 2.5 in the methanogenic archaea data sets, thereby corroborating a much more diverse eubacterial community. In both microbial groups, H-values started at lower initial values and became relatively stable after the first 2 weeks. The presence of abundant ARISA fragments from the archaea was comparably stable over the four major time phases (Fig. 4), although particular fragments (e.g. 713 bp), abundant in the earlier phases, disappeared in phase IV, while others (e.g. 747 bp) appeared in significant numbers only in later phases. In contrast to the methanogenic archaea, the total number of eubacterial ARISA fragments was considerably higher and the dominance of particular types as well as any tendency for (dis-)appearance was not observed (data not shown). This was further corroborated when the dominance among ARISA fragments was estimated by Simpson’s index for the different sampling groups. Within the eubacterial ARISA fragments Simpson’s index ‘1−λ’, as a measure for evenness, was close to 1, indicating that this fragment type distribution was hardly dominated by any specific fragment types. In contrast, a Simpson’s index of 0.8–0.9 was obtained for the ARISA fragments from the methanogenic archaea, indicated that some ARISA fragment types dominated within this group.

**Fig. 5:**
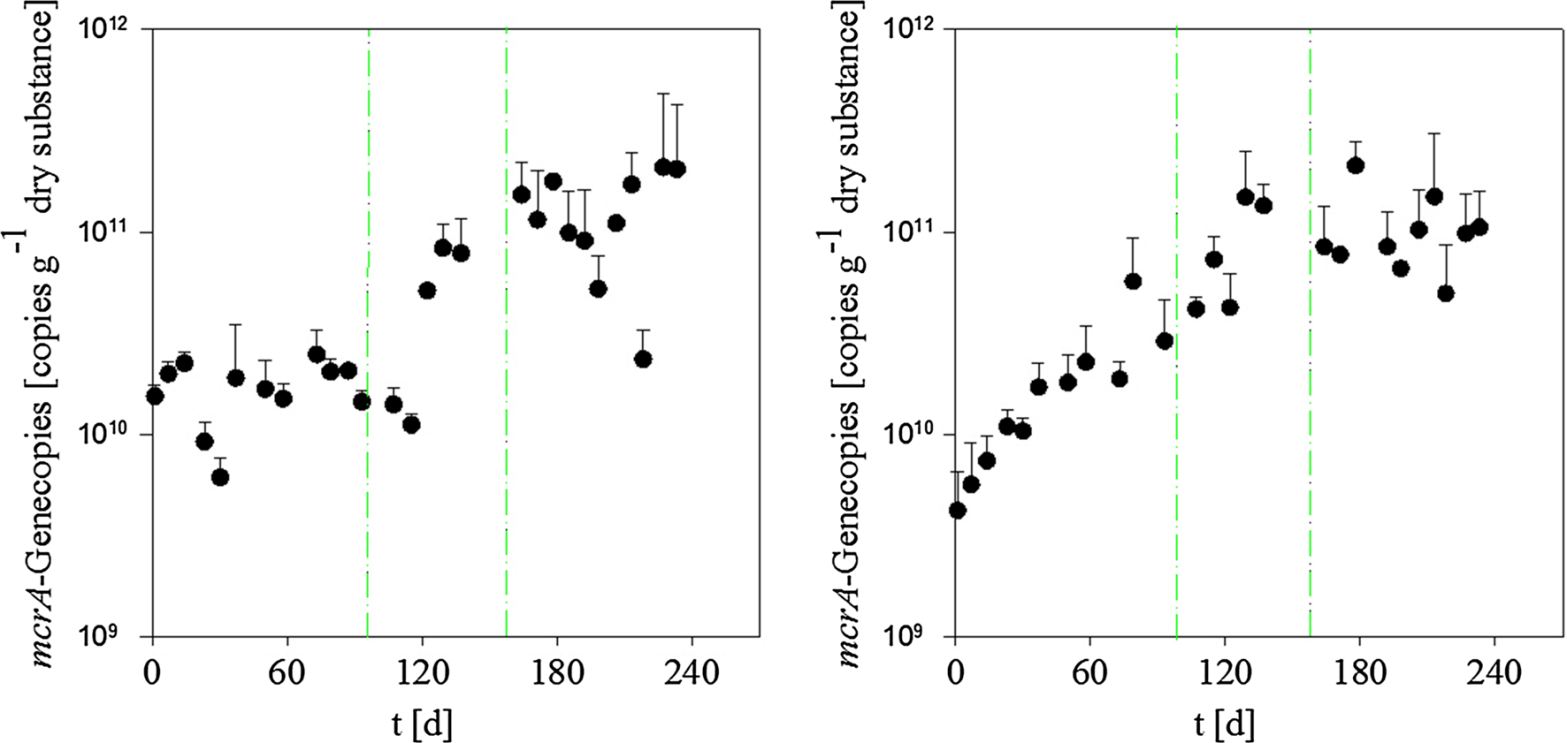
Development of the *mcrA* gene copy number in the fermenter (*left*) and the post digester (*right*) during the observation period. *Dash*-*dotted lines* mark feed changes. *First line* day 100, start of the addition of grass silage, *second line* day 160, start of the addition of ground wheat.

Finally, Fig. 5 shows the development of the total methanogen population in the two reactors in terms of the *mcrA* genes copy number. In the fermenter the *mcrA* gene copy number remained in the range of 10^10^ copies per gram dry substance for the first 90 days, while the *mcrA* gene copy number in the post digester increased from 4 × 10^9^ to 4 × 10^10^, i.e. by one order of magnitude, during that time. A comparison with Fig. 1 shows that this is not reflected by an increase in total biogas volume or methane content of the biogas in either compartment. Following day 90, save for a transitory dip in the fermenter, the *mcrA* gene copy number increased in both compartments over the next 70 days, reaching a stable number of approximately 10^11^ copies per gram dry substance, which was maintained throughout the remainder of the observation period.

**Fig. 6:**
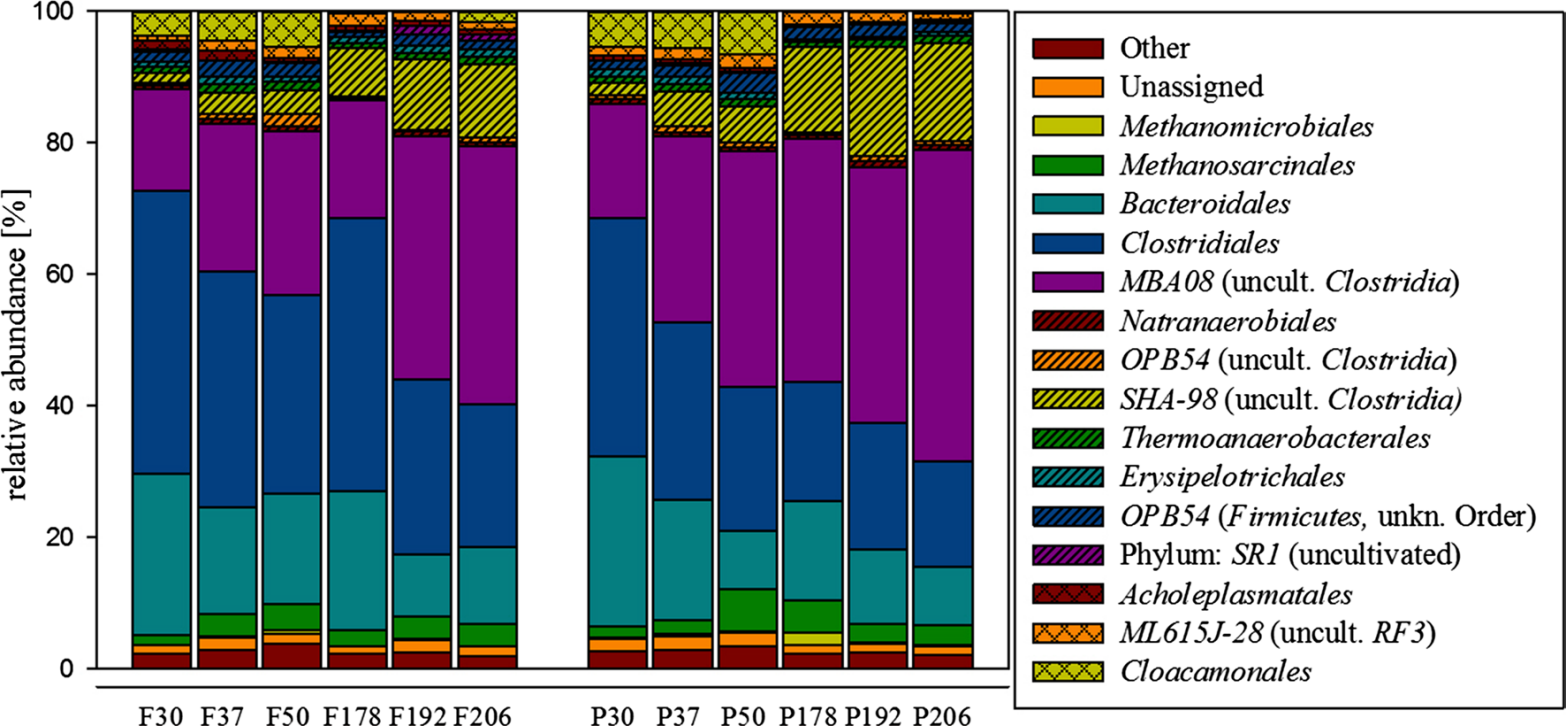
Relative abundance of 16S rRNA sequences at the order level in selected samples taken from the fermenter (F) and the post-digester (P), respectively, at the days indicated. Sequences appearing with an abundance of less than 1 % of the total are summarized and displayed as “others”. (Complete sequencing data at genus level is shown in Additional file 1)

Deep sequencing of selected fermenter and post-digester samples from time phases II and IV showed that about 94.7 % of the sequences could be assigned to the bacterial kingdom, while ca. 3.65 % were of archaeal origin. The remaining-small-portion could not be assigned to known taxa (Fig. 6). As expected, the majority of bacterial taxa were assigned to *Clostridiales* (including the uncultured orders *MBA08* and *SHA*-*98*), which are known to contain many anaerobic bacteria. Furthermore, the two archaeal orders of *Methanosarcinales* and *Methanomicrobiales* were identified as prominent archaeal groups present in the samples. While the abundance of most bacterial and archaeal orders did not vary greatly along the biogas plant run, one particular bacterial group seemed to be present in great number in phase 2 but at significantly lower level in the phase 4 samples. This order of uncultured *Cloacamonales* has been previously described as a new bacterial phylum *WWE1* branching deeply from the *Spirochaetes* and has been identified in municipal wastewater treatment plants (Pelletier et al. 2008; Chouari et al. 2005).

## Discussion

Automated ribosomal intergenic spacer analysis allows the simple and reliable analysis of microbial community structures. Provided identical primers are used, the method is easily standardized and then can serve as basis for comparative studies between different samples. Since absolute values for yield and effectivity of DNA isolation procedures from environmental samples are difficult to quantify with certainty, such insights into the relative differences derived under standardized experimental conditions are of particular value in environmental studies.

In this study ARISA was used as diagnostic tool to gain insight into the dynamics of biogas producing microbial communities. In this context, concerns have recently been raised about the reliability of commonly used primer systems (Purahong et al. 2015). In particular, the eubacterial primers (ITSF/ITSReub) used also in our ARISA study have come under critical investigation and several in silico amplified sequences were reported to represent nor microbial, but rather chloroplast sequences of plants, including *Sorghum, Oryza, Brassica* and several *Zea* species. However, careful inspection of the corresponding ITS regions of reference chloroplast genomes available at NCBI (http://www.ncbi.nlm.nih.gov/genome/) revealed that this requires at least one base pair mismatch. Furthermore, the length of these regions would be ca. 2.4 kb, which is much longer than the bacterial and archaeal regions to be expected (in our case successfully amplified fragments were, e.g. all <1000 bp). In addition, most of the putative chloroplast sequences reported in Purahong et al. (2015) were removed from NCBI sequence databases due to obvious contamination of the plant material with microorganisms. Therefore the ARISA and the NGS data of our study can with confidence be taken to describe mainly (if not exclusively) the prokaryotic community composition and dynamics of the investigated biogas reactors.

PCO analysis of the ARISA similarity matrices obtained for the “inoculation phase”, i.e. up to day 14, showed that significant differences existed for both the bacteria and the methanogenic archaea between the community structures of the fermenter and the post digester. Such differences between the two consecutive compartments were unexpected and show that the inoculation of bioreactors harboring complex communities is far from straightforward. Given that this is the first time the start up phase of an agricultural energy crop digester has been documented, further research is necessary, but it is quite possible that the “defined inoculum strategy” desired by many applicants is difficult to implement.

Following inoculation, ARISA allowed to divide the temporal development of the community structures into several distinct phases, intercepted by transition periods of 1–4 weeks. Contrarily to our expectations, changes in the community structures did not necessarily coincide with changes in the reactor performance on the macroscopic level nor could they always be explained by changes in the operational parameters. For instance, after the initial start-up phase, the biogas plant was stable for the first 100 days of operation in terms of biogas production/quality and abiotic process parameters, yet the microbial communities passed through phase II (23–58 days) and into phase III (73–137 days) during that time, a transition, which was thus silent on the macroscopic level. The fact that the community structures for both bacteria and archaea became similar in the two reactor compartments during that phase, was interpreted by us as a sign of stabilization of the microbial community.

From day 100 onwards, the by then established phase III communities were confronted with a change in feed, as grass silage was added to the corn silage. According to Lebuhn et al. (2014), grass silage has a higher content of micronutrients relevant for biogas production. In our case, the switch in feed composition resulted in a major disturbance of the process, indicated by dropping methane contents, together with a build up of acetic acid and eventually also H_2_ in both reactors, arguing for an inhibition of both acetoclastic and hydrogenotrophic methane production. On the level of the community structures, the eubacterial communities of fermenter and post digester started to deviate again (R value: 0.261, p = 0.017); a comparable separation was not detected within the archaeal ARISA fragments. What did change, however, was the metabolic activity of the archaea in the fermenter, where the *mcrA*-gene copy number decreased. This was not the case for the post digester. Taken together, this can be interpreted as a disturbance of the syntrophic relationship between the bacterial and the archaeal communities in the fermenter. Any change in feed will obviously affect first the bacterial community in the fermenter, which performs the initial degradation steps of the raw biomass. It is also possible that a new selection of eubacteria entered the fermenter with the grass silage, which is less likely in case of the strictly anaerobic archaeal methanogens.

While it took until day 137 before the statistical analysis of the community patterns indicated the end of phase III and entry into a period of transitorial change, the inability of the methanogenic archaea to metabolize the intermediates provided by the eubacteria put the communities in both reactors under stress already before that, in particular since the H_2_-concentrations was starting to rise 10 days after the introduction of the grass silage threatening to reach inhibitory levels. At that point the methanogenic community reacted, initially on a metabolic level. Between days 115 and 122, the copy number of the *mcrA*-genes increased rapidly, in particularly in the fermenter, where it augmented by 20 % from 1 × 10^10^ copies to 5 × 10^10^ copies per gram of dry substance. On the process level, this is characterized be a rapid increase in the methane content of the biogas, while the elevated VFA and H_2_ concentrations dropped.

However, the community structures were not stabilized by this reaction. Instead all four communities entered a transition period until day 164, at which point the biogas quality had stabilized at higher methane content than before (ca. 55 %) and a fourth type of eubacterial and methanogenic archaeal community structures had established itself. Moreover, structures of the eubacterial communities were now again similar in the reactor and post digester. The addition of ground wheat to the feed mixture at that point was not affecting either the community structures or the reactor performance.

Taken together ARISA, allows for the simple and reliable analysis and comparison of microbial community structures in biogas plants of technical dimensions. We found the method particularly useful as a diagnostic tool to follow the dynamic development of the microbial consortia in an agricultural biogas plant in terms of fragment number and quantity as well as their diversity and the possible dominance of the respective consortia by individual ARISA fragment types. In combination with high throughput sequencing of 16S rRNA regions from eubacteria and archaea, an in-depth metagenome analysis at broad taxonomic coverage were achieved. The development from the start-up phase to a stable working microbial community takes less than 30 days, while dynamic changes were occurring for more than 240 days.

## Additional file

10.1186/s13568-016-0219-7 Complete sequencing data at genus level.
