# Supplementary material for: Process parameters and changes in the microbial community patterns during the first 240 days of an agricultural energy crop digester
Source: AMB Express. 2016 Aug 2;6:53. doi: 10.1186/s13568-016-0219-7 (PMC4970986; doi:10.1186/s13568-016-0219-7)
Supplement: Supplementary file 1 — 10.1186/s13568-016-0219-7 Complete sequencing data at genus level. [file 13568_2016_219_MOESM1_ESM.pdf]

Process Parameters and changes in the microbial community patterns during the first 240 days of an agricultural energy crop digester

AMB Express

Authors: N.Weithmann, A.Weig, R. Freitag*

* corresponding author

Process Biotechnology, Center for Energy Technology, University of Bayreuth, 95440 Bayreuth, Germany

ruth.freitag@uni-bayreuth.de, Tel.: +49 921 557371

Taxa Summaries

Legend	Taxonomy	Total	F30	F37	F50	F178	F192	F206	P30	P37	P50	P178	P192	P206
		%	%	%	%	%	%	%	%	%	%	%	%	%
	Unassigned;Other;Other;Other;Other;Other	1.6%	1.2%	2.0%	1.6%	1.1%	1.9%	1.4%	1.9%	2.1%	2.1%	1.3%	1.4%	1.4%
	k__Archaea;p__Crenarchaeota;c__MCG;o__pGrfC26;f__;g__	0.0%	0.0%	0.0%	0.0%	0.0%	0.0%	0.0%	0.0%	0.0%	0.0%	0.0%	0.0%	0.0%
	k__Archaea;p__Euryarchaeota;c__Methanobacteria;o__Methanobacteriales;f__Methanobacteriaceae;g__Methanobacterium	0.0%	0.0%	0.0%	0.0%	0.0%	0.0%	0.0%	0.0%	0.0%	0.0%	0.0%	0.0%	0.0%
	k__Archaea;p__Euryarchaeota;c__Methanobacteria;o__Methanobacteriales;f__WSA2;g__	0.0%	0.0%	0.0%	0.0%	0.0%	0.0%	0.0%	0.0%	0.0%	0.1%	0.0%	0.0%	0.0%
	k__Archaea;p__Euryarchaeota;c__Methanomicrobia;o__Methanomicrobiales;f__Methanocorpusculaceae;g__Methanocorpusculum	0.0%	0.0%	0.0%	0.0%	0.0%	0.0%	0.0%	0.0%	0.0%	0.0%	0.0%	0.0%	0.0%
	k__Archaea;p__Euryarchaeota;c__Methanomicrobia;o__Methanomicrobiales;f__Methanomicrobiaceae;g__Methanoculleus	0.3%	0.1%	0.2%	0.5%	0.0%	0.1%	0.0%	0.2%	0.2%	0.2%	1.9%	0.1%	0.1%
	k__Archaea;p__Euryarchaeota;c__Methanomicrobia;o__Methanomicrobiales;f__Methanospirillaceae;g__Methanospirillum	0.0%	0.0%	0.0%	0.0%	0.0%	0.0%	0.0%	0.0%	0.0%	0.0%	0.0%	0.0%	0.0%
	k__Archaea;p__Euryarchaeota;c__Methanomicrobia;o__Methanosarcinales;Other;Other	0.0%	0.0%	0.0%	0.0%	0.0%	0.0%	0.0%	0.0%	0.0%	0.1%	0.0%	0.0%	0.0%
	k__Archaea;p__Euryarchaeota;c__Methanomicrobia;o__Methanosarcinales;f__Methanosaetaceae;g__Methanosaeta	0.8%	0.4%	0.9%	1.7%	0.0%	0.0%	0.0%	0.4%	1.6%	4.3%	0.0%	0.0%	0.0%
	k__Archaea;p__Euryarchaeota;c__Methanomicrobia;o__Methanosarcinales;f__Methanosarcinaceae;g__Methanosarcina	2.5%	1.0%	2.4%	2.3%	2.4%	3.5%	3.3%	1.2%	0.5%	2.1%	4.9%	2.8%	3.1%
	k__Archaea;p__Euryarchaeota;c__Methanomicrobia;o__YC-E6;f__;g__	0.0%	0.0%	0.0%	0.0%	0.0%	0.0%	0.0%	0.0%	0.0%	0.0%	0.0%	0.0%	0.0%
	k__Archaea;p__Euryarchaeota;c__Thermoplasmata;o__E2;f__[Methanomassiliicoccaceae];g__Methanomassiliicoccus	0.0%	0.0%	0.0%	0.0%	0.0%	0.0%	0.0%	0.0%	0.0%	0.1%	0.2%	0.0%	0.0%
	k__Archaea;p__Euryarchaeota;c__Thermoplasmata;o__E2;f__[Methanomassiliicoccaceae];g__vadinCA11	0.0%	0.0%	0.0%	0.0%	0.0%	0.0%	0.0%	0.0%	0.0%	0.0%	0.0%	0.0%	0.0%
	k__Bacteria;Other;Other;Other;Other;Other	0.0%	0.0%	0.0%	0.0%	0.0%	0.0%	0.0%	0.0%	0.0%	0.0%	0.0%	0.0%	0.0%
	k__Bacteria;p__;c__;o__;f__;g__	0.0%	0.0%	0.0%	0.0%	0.0%	0.0%	0.0%	0.0%	0.0%	0.0%	0.0%	0.0%	0.0%
	k__Bacteria;p__Acidobacteria;c__Acidobacteria-6;o__iii1-15;f__;g__	0.0%	0.0%	0.0%	0.0%	0.0%	0.0%	0.0%	0.0%	0.0%	0.0%	0.0%	0.0%	0.0%
	k__Bacteria;p__Acidobacteria;c__BPC102;o__MVS-40;f__;g__	0.0%	0.0%	0.0%	0.1%	0.0%	0.0%	0.0%	0.1%	0.0%	0.0%	0.0%	0.0%	0.0%
	k__Bacteria;p__Acidobacteria;c__Solibacteres;o__Solibacterales;f__AKIW659;g__	0.0%	0.0%	0.0%	0.0%	0.0%	0.0%	0.0%	0.0%	0.0%	0.0%	0.0%	0.0%	0.0%
	k__Bacteria;p__Acidobacteria;c__Solibacteres;o__Solibacterales;f__Solibacteraceae;g__Candidatus Solibacter	0.0%	0.0%	0.0%	0.0%	0.0%	0.0%	0.0%	0.0%	0.0%	0.0%	0.0%	0.0%	0.0%
	k__Bacteria;p__Actinobacteria;c__;o__;f__;g__	0.0%	0.0%	0.0%	0.0%	0.0%	0.0%	0.0%	0.0%	0.0%	0.0%	0.0%	0.0%	0.0%
	k__Bacteria;p__Actinobacteria;c__Acidimicrobiia;o__Acidimicrobiales;f__;g__	0.0%	0.0%	0.0%	0.0%	0.0%	0.0%	0.0%	0.0%	0.0%	0.0%	0.0%	0.0%	0.0%
	k__Bacteria;p__Actinobacteria;c__Actinobacteria;o__Actinomycetales;f__;g__	0.0%	0.0%	0.0%	0.0%	0.0%	0.0%	0.0%	0.0%	0.0%	0.0%	0.0%	0.0%	0.0%
	k__Bacteria;p__Actinobacteria;c__Actinobacteria;o__Actinomycetales;f__Actinomycetaceae;g__Actinomyces	0.0%	0.0%	0.0%	0.0%	0.0%	0.0%	0.0%	0.0%	0.0%	0.0%	0.0%	0.0%	0.0%
	k__Bacteria;p__Actinobacteria;c__Actinobacteria;o__Actinomycetales;f__Cellulomonadaceae;g__Actinotalea	0.0%	0.0%	0.0%	0.0%	0.0%	0.0%	0.0%	0.0%	0.0%	0.0%	0.0%	0.0%	0.0%
	k__Bacteria;p__Actinobacteria;c__Actinobacteria;o__Actinomycetales;f__Cellulomonadaceae;g__Cellulomonas	0.0%	0.0%	0.0%	0.0%	0.0%	0.0%	0.0%	0.0%	0.0%	0.0%	0.0%	0.0%	0.0%
	k__Bacteria;p__Actinobacteria;c__Actinobacteria;o__Actinomycetales;f__Corynebacteriaceae;g__Corynebacterium	0.0%	0.0%	0.0%	0.0%	0.0%	0.0%	0.0%	0.0%	0.0%	0.0%	0.0%	0.0%	0.0%
	k__Bacteria;p__Actinobacteria;c__Actinobacteria;o__Actinomycetales;f__Intrasporangiaceae;g__	0.0%	0.0%	0.0%	0.0%	0.0%	0.0%	0.0%	0.0%	0.0%	0.0%	0.0%	0.0%	0.0%
	k__Bacteria;p__Actinobacteria;c__Actinobacteria;o__Actinomycetales;f__Microbacteriaceae;Other	0.0%	0.0%	0.0%	0.0%	0.0%	0.0%	0.0%	0.0%	0.0%	0.0%	0.0%	0.0%	0.0%
	k__Bacteria;p__Actinobacteria;c__Actinobacteria;o__Actinomycetales;f__Microbacteriaceae;g__Salinibacterium	0.0%	0.0%	0.0%	0.0%	0.0%	0.0%	0.0%	0.0%	0.0%	0.0%	0.0%	0.0%	0.0%
	k__Bacteria;p__Actinobacteria;c__Actinobacteria;o__Actinomycetales;f__Micromonosporaceae;g__	0.0%	0.0%	0.0%	0.0%	0.0%	0.0%	0.0%	0.0%	0.0%	0.0%	0.0%	0.0%	0.0%
	k__Bacteria;p__Actinobacteria;c__Actinobacteria;o__Actinomycetales;f__Nocardiopsaceae;g__Thermobifida	0.0%	0.0%	0.0%	0.0%	0.0%	0.0%	0.0%	0.0%	0.0%	0.0%	0.0%	0.0%	0.0%
	k__Bacteria;p__Actinobacteria;c__Actinobacteria;o__Actinomycetales;f__Pseudonocardiaceae;g__	0.0%	0.0%	0.0%	0.0%	0.0%	0.0%	0.0%	0.0%	0.0%	0.0%	0.0%	0.0%	0.0%
	k__Bacteria;p__Actinobacteria;c__Actinobacteria;o__Actinomycetales;f__Pseudonocardiaceae;g__Saccharomonospora	0.0%	0.0%	0.0%	0.0%	0.0%	0.0%	0.0%	0.0%	0.0%	0.0%	0.0%	0.0%	0.0%
	k__Bacteria;p__Actinobacteria;c__Actinobacteria;o__Actinomycetales;f__Thermomonosporaceae;g__Actinomadura	0.0%	0.0%	0.0%	0.0%	0.0%	0.0%	0.0%	0.0%	0.0%	0.0%	0.0%	0.0%	0.0%
	k__Bacteria;p__Actinobacteria;c__Actinobacteria;o__Bifidobacteriales;f__Bifidobacteriaceae;g__Bifidobacterium	0.0%	0.0%	0.0%	0.0%	0.0%	0.0%	0.0%	0.0%	0.0%	0.0%	0.0%	0.0%	0.0%
	k__Bacteria;p__Actinobacteria;c__Actinobacteria;o__WCHB1-81;f__At425_EubF1;g__	0.0%	0.0%	0.0%	0.0%	0.0%	0.0%	0.0%	0.0%	0.0%	0.0%	0.0%	0.0%	0.0%
	k__Bacteria;p__Actinobacteria;c__Coriobacteriia;o__Coriobacteriales;f__Coriobacteriaceae;g__	0.0%	0.0%	0.0%	0.0%	0.0%	0.0%	0.0%	0.0%	0.0%	0.0%	0.0%	0.0%	0.0%
	k__Bacteria;p__Actinobacteria;c__Thermoleophilia;o__;f__;g__	0.0%	0.0%	0.0%	0.0%	0.0%	0.0%	0.0%	0.0%	0.0%	0.0%	0.0%	0.0%	0.0%
	k__Bacteria;p__Actinobacteria;c__Thermoleophilia;o__Gaiellales;f__;g__	0.0%	0.0%	0.0%	0.0%	0.0%	0.0%	0.0%	0.0%	0.0%	0.0%	0.0%	0.0%	0.0%
	k__Bacteria;p__Armatimonadetes;c__SJA-176;o__RB046;f__;g__	0.0%	0.0%	0.0%	0.0%	0.0%	0.0%	0.0%	0.0%	0.0%	0.0%	0.0%	0.0%	0.0%
	k__Bacteria;p__BRC1;c__NPL-UPA2;o__;f__;g__	0.0%	0.0%	0.0%	0.0%	0.0%	0.0%	0.0%	0.0%	0.0%	0.0%	0.0%	0.0%	0.0%
	k__Bacteria;p__BRC1;c__PRR-11;o__;f__;g__	0.0%	0.0%	0.0%	0.0%	0.0%	0.0%	0.0%	0.0%	0.0%	0.0%	0.0%	0.0%	0.0%
	k__Bacteria;p__Bacteroidetes;c__Bacteroidia;o__Bacteroidales;Other;Other	0.0%	0.0%	0.0%	0.0%	0.0%	0.0%	0.0%	0.0%	0.0%	0.0%	0.0%	0.0%	0.0%
	k__Bacteria;p__Bacteroidetes;c__Bacteroidia;o__Bacteroidales;f__;g__	6.4%	16.6%	6.6%	4.1%	9.5%	2.2%	3.6%	17.4%	2.8%	1.6%	7.2%	3.2%	2.3%
	k__Bacteria;p__Bacteroidetes;c__Bacteroidia;o__Bacteroidales;f__BA008;g__	0.0%	0.0%	0.0%	0.0%	0.0%	0.0%	0.0%	0.0%	0.0%	0.0%	0.0%	0.0%	0.0%
	k__Bacteria;p__Bacteroidetes;c__Bacteroidia;o__Bacteroidales;f__Bacteroidaceae;Other	0.0%	0.0%	0.0%	0.0%	0.0%	0.0%	0.0%	0.0%	0.0%	0.0%	0.0%	0.0%	0.0%
	k__Bacteria;p__Bacteroidetes;c__Bacteroidia;o__Bacteroidales;f__Bacteroidaceae;g__	0.7%	0.5%	0.9%	0.5%	0.9%	0.4%	0.3%	0.8%	1.2%	1.0%	0.9%	0.4%	0.2%
	k__Bacteria;p__Bacteroidetes;c__Bacteroidia;o__Bacteroidales;f__Bacteroidaceae;g__BF311	2.0%	2.0%	2.0%	2.0%	2.0%	2.0%	1.7%	2.5%	2.2%	2.0%	2.6%	1.7%	1.1%
	k__Bacteria;p__Bacteroidetes;c__Bacteroidia;o__Bacteroidales;f__Bacteroidaceae;g__Bacteroides	0.0%	0.0%	0.0%	0.0%	0.0%	0.0%	0.0%	0.0%	0.0%	0.0%	0.0%	0.0%	0.0%
	k__Bacteria;p__Bacteroidetes;c__Bacteroidia;o__Bacteroidales;f__Marinilabiaceae;Other	0.0%	0.0%	0.0%	0.0%	0.0%	0.0%	0.0%	0.0%	0.0%	0.0%	0.0%	0.0%	0.0%
	k__Bacteria;p__Bacteroidetes;c__Bacteroidia;o__Bacteroidales;f__Marinilabiaceae;g__	2.2%	1.8%	2.8%	7.1%	1.6%	0.5%	1.0%	0.7%	8.5%	1.5%	0.4%	0.5%	0.3%
	k__Bacteria;p__Bacteroidetes;c__Bacteroidia;o__Bacteroidales;f__Porphyromonadaceae;Other	0.0%	0.0%	0.0%	0.0%	0.0%	0.0%	0.0%	0.0%	0.0%	0.0%	0.0%	0.0%	0.0%
	k__Bacteria;p__Bacteroidetes;c__Bacteroidia;o__Bacteroidales;f__Porphyromonadaceae;g__	4.0%	3.4%	3.5%	2.6%	6.4%	4.1%	4.9%	4.2%	3.0%	2.7%	3.6%	5.3%	4.8%
	k__Bacteria;p__Bacteroidetes;c__Bacteroidia;o__Bacteroidales;f__Porphyromonadaceae;g__Paludibacter	0.3%	0.2%	0.4%	0.3%	0.8%	0.2%	0.2%	0.2%	0.7%	0.2%	0.4%	0.2%	0.1%
	k__Bacteria;p__Bacteroidetes;c__Bacteroidia;o__Bacteroidales;f__Porphyromonadaceae;g__Parabacteroides	0.0%	0.0%	0.0%	0.0%	0.0%	0.0%	0.0%	0.0%	0.0%	0.0%	0.0%	0.0%	0.0%
	k__Bacteria;p__Bacteroidetes;c__Bacteroidia;o__Bacteroidales;f__Prevotellaceae;g__	0.0%	0.0%	0.0%	0.0%	0.0%	0.0%	0.0%	0.0%	0.0%	0.0%	0.0%	0.0%	0.0%
	k__Bacteria;p__Bacteroidetes;c__Bacteroidia;o__Bacteroidales;f__SB-1;g__	0.0%	0.0%	0.0%	0.0%	0.0%	0.0%	0.0%	0.0%	0.0%	0.0%	0.0%	0.0%	0.0%
	k__Bacteria;p__Bacteroidetes;c__Bacteroidia;o__Bacteroidales;f__[Paraprevotellaceae];g__	0.0%	0.0%	0.0%	0.0%	0.0%	0.0%	0.0%	0.0%	0.0%	0.0%	0.0%	0.0%	0.0%
	k__Bacteria;p__Bacteroidetes;c__Sphingobacteriia;o__Sphingobacteriales;f__Sphingobacteriaceae;g__	0.0%	0.0%	0.0%	0.0%	0.0%	0.0%	0.0%	0.0%	0.0%	0.0%	0.0%	0.0%	0.0%
	k__Bacteria;p__Bacteroidetes;c__Sphingobacteriia;o__Sphingobacteriales;f__Sphingobacteriaceae;g__Sphingobacterium	0.0%	0.0%	0.0%	0.0%	0.0%	0.0%	0.0%	0.0%	0.0%	0.0%	0.0%	0.0%	0.0%
	k__Bacteria;p__Chloroflexi;c__Anaerolineae;o__;f__;g__	0.0%	0.0%	0.0%	0.1%	0.0%	0.0%	0.0%	0.1%	0.0%	0.0%	0.0%	0.0%	0.0%
	k__Bacteria;p__Chloroflexi;c__Anaerolineae;o__Anaerolineales;f__Anaerolinaceae;g__Anaerolinea	0.0%	0.0%	0.0%	0.0%	0.0%	0.0%	0.0%	0.0%	0.0%	0.0%	0.0%	0.0%	0.0%
	k__Bacteria;p__Chloroflexi;c__Anaerolineae;o__Anaerolineales;f__Anaerolinaceae;g__SHD-231	0.0%	0.0%	0.0%	0.0%	0.0%	0.0%	0.0%	0.0%	0.0%	0.0%	0.0%	0.0%	0.0%
	k__Bacteria;p__Chloroflexi;c__Anaerolineae;o__Anaerolineales;f__Anaerolinaceae;g__T78	0.0%	0.0%	0.0%	0.0%	0.0%	0.0%	0.0%	0.0%	0.0%	0.0%	0.0%	0.0%	0.0%
	k__Bacteria;p__Chloroflexi;c__Anaerolineae;o__CFB-26;f__;g__	0.0%	0.0%	0.0%	0.0%	0.0%	0.0%	0.0%	0.0%	0.0%	0.0%	0.0%	0.0%	0.0%
	k__Bacteria;p__Chloroflexi;c__Anaerolineae;o__Caldilineales;f__Caldilineaceae;g__	0.0%	0.0%	0.0%	0.0%	0.0%	0.0%	0.0%	0.0%	0.0%	0.0%	0.0%	0.0%	0.0%
	k__Bacteria;p__Chloroflexi;c__Anaerolineae;o__GCA004;f__;g__	0.0%	0.0%	0.0%	0.0%	0.0%	0.0%	0.0%	0.0%	0.0%	0.0%	0.0%	0.0%	0.0%
	k__Bacteria;p__Chloroflexi;c__Anaerolineae;o__SBR1031;f__SHA-31;g__	0.0%	0.0%	0.0%	0.0%	0.0%	0.0%	0.0%	0.0%	0.0%	0.0%	0.0%	0.0%	0.0%
	k__Bacteria;p__Chloroflexi;c__Anaerolineae;o__SHA-20;f__;g__	0.0%	0.0%	0.0%	0.0%	0.0%	0.0%	0.0%	0.0%	0.0%	0.0%	0.0%	0.0%	0.0%
	k__Bacteria;p__Chloroflexi;c__Dehalococcoidetes;o__Dehalococcoidales;f__Dehalococcoidaceae;g__	0.0%	0.0%	0.0%	0.0%	0.0%	0.0%	0.0%	0.0%	0.0%	0.0%	0.0%	0.0%	0.0%
	k__Bacteria;p__Chloroflexi;c__SHA-26;o__;f__;g__	0.0%	0.0%	0.0%	0.0%	0.0%	0.0%	0.0%	0.0%	0.0%	0.0%	0.0%	0.0%	0.0%
	k__Bacteria;p__Chloroflexi;c__TK10;o__;f__;g__	0.0%	0.0%	0.0%	0.0%	0.0%	0.0%	0.0%	0.0%	0.0%	0.0%	0.0%	0.0%	0.0%
	k__Bacteria;p__Chloroflexi;c__Thermomicrobia;o__JG30-KF-CM45;f__;g__	0.0%	0.0%	0.0%	0.0%	0.0%	0.0%	0.0%	0.0%	0.0%	0.0%	0.0%	0.0%	0.0%
	k__Bacteria;p__Chloroflexi;c__Thermomicrobia;o__Sphaerobacterales;f__;g__	0.0%	0.0%	0.0%	0.0%	0.0%	0.0%	0.0%	0.0%	0.0%	0.0%	0.0%	0.0%	0.0%
	k__Bacteria;p__Cyanobacteria;c__4C0d-2;o__YS2;f__;g__	0.0%	0.0%	0.0%	0.0%	0.0%	0.0%	0.0%	0.0%	0.0%	0.0%	0.0%	0.0%	0.0%
	k__Bacteria;p__Cyanobacteria;c__Chloroplast;o__Streptophyta;f__;g__	0.0%	0.0%	0.0%	0.0%	0.0%	0.0%	0.0%	0.0%	0.0%	0.0%	0.0%	0.0%	0.0%
	k__Bacteria;p__Fibrobacteres;c__Fibrobacteria;o__Fibrobacterales;f__;g__	0.0%	0.0%	0.0%	0.0%	0.0%	0.0%	0.0%	0.0%	0.0%	0.0%	0.0%	0.0%	0.0%
	k__Bacteria;p__Fibrobacteres;c__Fibrobacteria;o__Fibrobacterales;f__Fibrobacteraceae;g__Fibrobacter	0.0%	0.0%	0.0%	0.0%	0.0%	0.0%	0.0%	0.0%	0.0%	0.0%	0.0%	0.0%	0.0%
	k__Bacteria;p__Fibrobacteres;c__TG3;o__TG3-1;f__TSCOR003-O20;g__	0.2%	0.2%	0.3%	0.7%	0.0%	0.0%	0.0%	0.4%	0.4%	0.2%	0.0%	0.0%	0.0%
	k__Bacteria;p__Firmicutes;Other;Other;Other;Other	0.0%	0.0%	0.0%	0.0%	0.0%	0.0%	0.0%	0.0%	0.0%	0.0%	0.0%	0.0%	0.0%
	k__Bacteria;p__Firmicutes;c__Bacilli;o__Bacillales;f__;g__	0.0%	0.0%	0.0%	0.0%	0.0%	0.0%	0.0%	0.0%	0.0%	0.0%	0.0%	0.0%	0.0%
	k__Bacteria;p__Firmicutes;c__Bacilli;o__Bacillales;f__Bacillaceae;g__	0.0%	0.0%	0.0%	0.0%	0.0%	0.0%	0.0%	0.0%	0.0%	0.0%	0.0%	0.0%	0.0%
	k__Bacteria;p__Firmicutes;c__Bacilli;o__Bacillales;f__Bacillaceae;g__Bacillus	0.0%	0.0%	0.0%	0.0%	0.0%	0.0%	0.0%	0.0%	0.0%	0.0%	0.0%	0.0%	0.0%
	k__Bacteria;p__Firmicutes;c__Bacilli;o__Bacillales;f__Paenibacillaceae;g__	0.0%	0.0%	0.0%	0.0%	0.0%	0.0%	0.0%	0.0%	0.0%	0.0%	0.0%	0.0%	0.0%
	k__Bacteria;p__Firmicutes;c__Bacilli;o__Bacillales;f__Paenibacillaceae;g__Brevibacillus	0.0%	0.0%	0.0%	0.0%	0.0%	0.0%	0.0%	0.0%	0.0%	0.0%	0.0%	0.0%	0.0%
	k__Bacteria;p__Firmicutes;c__Bacilli;o__Bacillales;f__Paenibacillaceae;g__Paenibacillus	0.0%	0.0%	0.0%	0.0%	0.0%	0.0%	0.0%	0.0%	0.0%	0.0%	0.0%	0.0%	0.0%
	k__Bacteria;p__Firmicutes;c__Bacilli;o__Bacillales;f__Planococcaceae;Other	0.0%	0.0%	0.0%	0.0%	0.0%	0.0%	0.0%	0.0%	0.0%	0.0%	0.0%	0.0%	0.0%
	k__Bacteria;p__Firmicutes;c__Bacilli;o__Bacillales;f__Planococcaceae;g__	0.0%	0.0%	0.0%	0.0%	0.0%	0.0%	0.0%	0.0%	0.0%	0.0%	0.0%	0.0%	0.0%
	k__Bacteria;p__Firmicutes;c__Bacilli;o__Bacillales;f__Planococcaceae;g__Lysinibacillus	0.0%	0.0%	0.0%	0.0%	0.0%	0.0%	0.0%	0.0%	0.0%	0.0%	0.0%	0.0%	0.0%
	k__Bacteria;p__Firmicutes;c__Bacilli;o__Bacillales;f__Planococcaceae;g__Planococcus	0.0%	0.0%	0.0%	0.0%	0.0%	0.0%	0.0%	0.0%	0.0%	0.0%	0.0%	0.0%	0.0%
	k__Bacteria;p__Firmicutes;c__Bacilli;o__Bacillales;f__Planococcaceae;g__Rummeliibacillus	0.0%	0.0%	0.0%	0.0%	0.0%	0.0%	0.0%	0.0%	0.0%	0.0%	0.0%	0.0%	0.0%
	k__Bacteria;p__Firmicutes;c__Bacilli;o__Bacillales;f__Planococcaceae;g__Ureibacillus	0.0%	0.0%	0.0%	0.0%	0.0%	0.0%	0.0%	0.0%	0.0%	0.0%	0.0%	0.0%	0.0%
	k__Bacteria;p__Firmicutes;c__Bacilli;o__Bacillales;f__Staphylococcaceae;g__Jeotgalicoccus	0.0%	0.0%	0.0%	0.0%	0.0%	0.0%	0.0%	0.0%	0.0%	0.0%	0.0%	0.0%	0.0%
	k__Bacteria;p__Firmicutes;c__Bacilli;o__Bacillales;f__Thermoactinomycetaceae;g__	0.0%	0.0%	0.0%	0.0%	0.0%	0.0%	0.0%	0.0%	0.0%	0.0%	0.0%	0.0%	0.0%
	k__Bacteria;p__Firmicutes;c__Bacilli;o__Bacillales;f__Thermoactinomycetaceae;g__Planifilum	0.0%	0.0%	0.0%	0.0%	0.0%	0.0%	0.0%	0.0%	0.0%	0.0%	0.0%	0.0%	0.0%
	k__Bacteria;p__Firmicutes;c__Bacilli;o__Haloplasmatales;f__Haloplasmataceae;g__	0.0%	0.0%	0.0%	0.0%	0.0%	0.0%	0.0%	0.0%	0.0%	0.0%	0.0%	0.0%	0.0%
	k__Bacteria;p__Firmicutes;c__Bacilli;o__Lactobacillales;f__;g__	0.0%	0.0%	0.0%	0.0%	0.0%	0.0%	0.0%	0.0%	0.0%	0.0%	0.0%	0.0%	0.0%

Taxa Summaries

	k__Bacteria;p__Firmicutes;c__Bacilli;o__Lactobacillales;f__Aerococcaceae;g__	0.0%	0.0%	0.0%	0.0%	0.0%	0.0%	0.0%	0.0%	0.0%	0.0%	0.0%	0.0%	0.0%
	k__Bacteria;p__Firmicutes;c__Bacilli;o__Lactobacillales;f__Aerococcaceae;g__Facklamia	0.0%	0.0%	0.0%	0.0%	0.0%	0.0%	0.0%	0.0%	0.0%	0.0%	0.0%	0.0%	0.0%
	k__Bacteria;p__Firmicutes;c__Bacilli;o__Lactobacillales;f__Enterococcaceae;g__Enterococcus	0.0%	0.0%	0.0%	0.0%	0.1%	0.0%	0.0%	0.0%	0.1%	0.1%	0.0%	0.0%	0.0%
	k__Bacteria;p__Firmicutes;c__Bacilli;o__Lactobacillales;f__Lactobacillaceae;g__Lactobacillus	0.0%	0.0%	0.0%	0.0%	0.0%	0.0%	0.0%	0.0%	0.0%	0.0%	0.0%	0.0%	0.0%
	k__Bacteria;p__Firmicutes;c__Bacilli;o__Lactobacillales;f__Lactobacillaceae;g__Pediococcus	0.0%	0.0%	0.0%	0.0%	0.0%	0.0%	0.0%	0.0%	0.0%	0.0%	0.0%	0.0%	0.0%
	k__Bacteria;p__Firmicutes;c__Bacilli;o__Lactobacillales;f__Streptococcaceae;g__Streptococcus	0.3%	0.3%	0.3%	0.3%	0.8%	0.4%	0.2%	0.2%	0.1%	0.3%	0.2%	0.4%	0.2%
	k__Bacteria;p__Firmicutes;c__Bacilli;o__Turicibacterales;f__Turicibacteraceae;g__Turicibacter	0.0%	0.0%	0.0%	0.0%	0.0%	0.0%	0.0%	0.0%	0.0%	0.0%	0.0%	0.0%	0.0%
	k__Bacteria;p__Firmicutes;c__Clostridia;Other;Other;Other	0.3%	0.1%	0.2%	0.2%	0.1%	0.5%	0.4%	0.1%	0.3%	0.4%	0.5%	0.7%	0.7%
	k__Bacteria;p__Firmicutes;c__Clostridia;o__;f__;g__	0.2%	0.2%	0.3%	0.3%	0.1%	0.2%	0.1%	0.3%	0.3%	0.3%	0.2%	0.2%	0.2%
	k__Bacteria;p__Firmicutes;c__Clostridia;o__BSA2B-08;f__;g__	0.2%	0.1%	0.2%	0.1%	0.1%	0.2%	0.2%	0.1%	0.1%	0.2%	0.2%	0.2%	0.2%
	k__Bacteria;p__Firmicutes;c__Clostridia;o__Clostridiales;Other;Other	0.2%	0.2%	0.2%	0.2%	0.2%	0.2%	0.2%	0.2%	0.2%	0.2%	0.1%	0.2%	0.2%
	k__Bacteria;p__Firmicutes;c__Clostridia;o__Clostridiales;f__;g__	0.6%	0.6%	0.9%	0.8%	0.8%	0.5%	0.4%	0.8%	0.8%	0.7%	0.6%	0.3%	0.2%
	k__Bacteria;p__Firmicutes;c__Clostridia;o__Clostridiales;f__Caldicoprobacteraceae;g__Caldicoprobacter	2.7%	3.6%	2.9%	2.9%	4.1%	2.6%	1.9%	3.8%	2.8%	2.2%	2.0%	2.4%	1.6%
	k__Bacteria;p__Firmicutes;c__Clostridia;o__Clostridiales;f__Christensenellaceae;g__	0.2%	0.3%	0.3%	0.2%	0.4%	0.2%	0.3%	0.2%	0.2%	0.1%	0.2%	0.3%	0.2%
	k__Bacteria;p__Firmicutes;c__Clostridia;o__Clostridiales;f__Clostridiaceae;Other	0.0%	0.0%	0.0%	0.0%	0.0%	0.0%	0.0%	0.0%	0.0%	0.0%	0.0%	0.0%	0.0%
	k__Bacteria;p__Firmicutes;c__Clostridia;o__Clostridiales;f__Clostridiaceae;g__	0.8%	1.0%	0.9%	0.7%	1.8%	1.1%	1.3%	0.8%	0.6%	0.4%	0.5%	0.5%	0.5%
	k__Bacteria;p__Firmicutes;c__Clostridia;o__Clostridiales;f__Clostridiaceae;g__02d06	0.0%	0.0%	0.0%	0.0%	0.0%	0.0%	0.0%	0.0%	0.0%	0.0%	0.0%	0.0%	0.0%
	k__Bacteria;p__Firmicutes;c__Clostridia;o__Clostridiales;f__Clostridiaceae;g__Alkaliphilus	0.0%	0.0%	0.0%	0.0%	0.0%	0.0%	0.0%	0.0%	0.0%	0.0%	0.0%	0.0%	0.0%
	k__Bacteria;p__Firmicutes;c__Clostridia;o__Clostridiales;f__Clostridiaceae;g__Clostridium	7.4%	15.8%	9.7%	8.3%	11.1%	6.4%	4.8%	9.5%	6.5%	4.3%	3.8%	5.2%	3.5%
	k__Bacteria;p__Firmicutes;c__Clostridia;o__Clostridiales;f__Clostridiaceae;g__Geosporobacter_Thermotalea	0.0%	0.0%	0.0%	0.0%	0.0%	0.0%	0.0%	0.0%	0.0%	0.0%	0.0%	0.0%	0.0%
	k__Bacteria;p__Firmicutes;c__Clostridia;o__Clostridiales;f__Clostridiaceae;g__Natronincola_Anaerovirgula	0.0%	0.0%	0.0%	0.0%	0.0%	0.0%	0.0%	0.0%	0.0%	0.0%	0.0%	0.0%	0.0%
	k__Bacteria;p__Firmicutes;c__Clostridia;o__Clostridiales;f__Dehalobacteriaceae;g__Dehalobacterium	0.1%	0.1%	0.1%	0.1%	0.1%	0.1%	0.1%	0.1%	0.1%	0.0%	0.0%	0.1%	0.1%
	k__Bacteria;p__Firmicutes;c__Clostridia;o__Clostridiales;f__EtOH8;g__	0.6%	0.8%	1.7%	0.6%	0.7%	0.6%	0.5%	0.4%	0.4%	0.3%	0.2%	0.3%	0.3%
	k__Bacteria;p__Firmicutes;c__Clostridia;o__Clostridiales;f__Eubacteriaceae;g__	0.0%	0.0%	0.0%	0.0%	0.0%	0.0%	0.0%	0.0%	0.0%	0.0%	0.0%	0.0%	0.0%
	k__Bacteria;p__Firmicutes;c__Clostridia;o__Clostridiales;f__Eubacteriaceae;g__Anaerofustis	0.0%	0.0%	0.0%	0.0%	0.0%	0.0%	0.0%	0.0%	0.0%	0.0%	0.0%	0.0%	0.0%
	k__Bacteria;p__Firmicutes;c__Clostridia;o__Clostridiales;f__Eubacteriaceae;g__Garciella	0.1%	0.1%	0.1%	0.2%	0.0%	0.0%	0.0%	0.2%	0.1%	0.1%	0.0%	0.0%	0.0%
	k__Bacteria;p__Firmicutes;c__Clostridia;o__Clostridiales;f__Eubacteriaceae;g__Pseudoramibacter_Eubacterium	0.0%	0.0%	0.0%	0.0%	0.0%	0.0%	0.0%	0.0%	0.0%	0.0%	0.0%	0.0%	0.0%
	k__Bacteria;p__Firmicutes;c__Clostridia;o__Clostridiales;f__Gracilibacteraceae;Other	0.0%	0.0%	0.0%	0.0%	0.0%	0.0%	0.0%	0.0%	0.0%	0.0%	0.0%	0.0%	0.0%
	k__Bacteria;p__Firmicutes;c__Clostridia;o__Clostridiales;f__Gracilibacteraceae;g__	0.1%	0.1%	0.0%	0.2%	0.0%	0.0%	0.0%	0.1%	0.1%	0.0%	0.0%	0.0%	0.0%
	k__Bacteria;p__Firmicutes;c__Clostridia;o__Clostridiales;f__Gracilibacteraceae;g__Gracilibacter	0.0%	0.1%	0.1%	0.1%	0.0%	0.0%	0.0%	0.0%	0.1%	0.0%	0.0%	0.0%	0.0%
	k__Bacteria;p__Firmicutes;c__Clostridia;o__Clostridiales;f__Gracilibacteraceae;g__Lutispora	0.1%	0.1%	0.1%	0.2%	0.2%	0.1%	0.1%	0.1%	0.1%	0.1%	0.1%	0.2%	0.1%
	k__Bacteria;p__Firmicutes;c__Clostridia;o__Clostridiales;f__Lachnospiraceae;Other	0.0%	0.1%	0.1%	0.0%	0.0%	0.1%	0.0%	0.0%	0.1%	0.0%	0.0%	0.0%	0.0%
	k__Bacteria;p__Firmicutes;c__Clostridia;o__Clostridiales;f__Lachnospiraceae;g__	1.1%	2.4%	2.3%	0.8%	2.2%	1.1%	0.6%	1.0%	1.5%	0.5%	0.3%	0.5%	0.2%
	k__Bacteria;p__Firmicutes;c__Clostridia;o__Clostridiales;f__Lachnospiraceae;g__Butyrivibrio	0.0%	0.0%	0.0%	0.0%	0.0%	0.0%	0.0%	0.0%	0.0%	0.0%	0.0%	0.0%	0.0%
	k__Bacteria;p__Firmicutes;c__Clostridia;o__Clostridiales;f__Lachnospiraceae;g__Coprococcus	0.9%	1.2%	1.4%	1.8%	1.7%	0.7%	0.6%	0.7%	1.7%	0.5%	0.3%	0.3%	0.2%
	k__Bacteria;p__Firmicutes;c__Clostridia;o__Clostridiales;f__Lachnospiraceae;g__Defluviitalea	0.0%	0.0%	0.0%	0.0%	0.0%	0.0%	0.0%	0.0%	0.0%	0.0%	0.0%	0.0%	0.0%
	k__Bacteria;p__Firmicutes;c__Clostridia;o__Clostridiales;f__Lachnospiraceae;g__Dorea	0.0%	0.0%	0.0%	0.0%	0.0%	0.0%	0.0%	0.0%	0.0%	0.0%	0.0%	0.0%	0.0%
	k__Bacteria;p__Firmicutes;c__Clostridia;o__Clostridiales;f__Lachnospiraceae;g__Lachnospira	0.0%	0.0%	0.0%	0.0%	0.0%	0.0%	0.0%	0.0%	0.0%	0.0%	0.0%	0.0%	0.0%
	k__Bacteria;p__Firmicutes;c__Clostridia;o__Clostridiales;f__Lachnospiraceae;g__[Ruminococcus]	0.0%	0.0%	0.0%	0.0%	0.0%	0.0%	0.0%	0.0%	0.0%	0.0%	0.0%	0.0%	0.0%
	k__Bacteria;p__Firmicutes;c__Clostridia;o__Clostridiales;f__Peptococcaceae;Other	0.0%	0.0%	0.0%	0.0%	0.0%	0.0%	0.0%	0.0%	0.0%	0.0%	0.0%	0.0%	0.0%
	k__Bacteria;p__Firmicutes;c__Clostridia;o__Clostridiales;f__Peptococcaceae;g__	0.4%	0.6%	0.6%	0.5%	0.5%	0.4%	0.3%	0.5%	0.4%	0.3%	0.2%	0.3%	0.2%
	k__Bacteria;p__Firmicutes;c__Clostridia;o__Clostridiales;f__Peptococcaceae;g__Dehalobacter_Syntrophobotulus	0.1%	0.1%	0.1%	0.1%	0.1%	0.1%	0.1%	0.1%	0.0%	0.0%	0.0%	0.1%	0.1%
	k__Bacteria;p__Firmicutes;c__Clostridia;o__Clostridiales;f__Peptococcaceae;g__Desulfitobacter	0.0%	0.0%	0.0%	0.0%	0.1%	0.0%	0.0%	0.0%	0.0%	0.0%	0.0%	0.0%	0.0%
	k__Bacteria;p__Firmicutes;c__Clostridia;o__Clostridiales;f__Peptococcaceae;g__Desulfosporosinus	0.0%	0.0%	0.0%	0.0%	0.0%	0.0%	0.0%	0.0%	0.0%	0.0%	0.0%	0.0%	0.0%
	k__Bacteria;p__Firmicutes;c__Clostridia;o__Clostridiales;f__Peptococcaceae;g__Desulfotomaculum	0.0%	0.0%	0.0%	0.0%	0.0%	0.0%	0.0%	0.0%	0.0%	0.0%	0.0%	0.0%	0.0%
	k__Bacteria;p__Firmicutes;c__Clostridia;o__Clostridiales;f__Peptococcaceae;g__Desulfotomaculum_Desulfovirgula	0.0%	0.0%	0.0%	0.0%	0.0%	0.0%	0.0%	0.0%	0.0%	0.0%	0.0%	0.0%	0.0%
	k__Bacteria;p__Firmicutes;c__Clostridia;o__Clostridiales;f__Peptococcaceae;g__Desulfurispora	0.0%	0.0%	0.0%	0.0%	0.0%	0.0%	0.0%	0.0%	0.0%	0.0%	0.0%	0.0%	0.0%
	k__Bacteria;p__Firmicutes;c__Clostridia;o__Clostridiales;f__Peptococcaceae;g__Pelotomaculum	0.3%	0.2%	0.3%	0.3%	0.5%	0.4%	0.3%	0.4%	0.2%	0.3%	0.2%	0.2%	0.3%
	k__Bacteria;p__Firmicutes;c__Clostridia;o__Clostridiales;f__Peptococcaceae;g__Sporotomaculum	0.0%	0.0%	0.0%	0.0%	0.0%	0.0%	0.0%	0.0%	0.0%	0.0%	0.0%	0.0%	0.0%
	k__Bacteria;p__Firmicutes;c__Clostridia;o__Clostridiales;f__Peptostreptococcaceae;g__	0.0%	0.0%	0.0%	0.0%	0.0%	0.0%	0.0%	0.0%	0.0%	0.0%	0.0%	0.0%	0.0%
	k__Bacteria;p__Firmicutes;c__Clostridia;o__Clostridiales;f__Ruminococcaceae;Other	0.0%	0.0%	0.0%	0.0%	0.0%	0.0%	0.0%	0.0%	0.0%	0.0%	0.0%	0.0%	0.0%
	k__Bacteria;p__Firmicutes;c__Clostridia;o__Clostridiales;f__Ruminococcaceae;g__	4.6%	5.6%	5.5%	5.6%	5.4%	3.9%	3.3%	5.8%	5.1%	3.9%	4.0%	4.0%	2.8%
	k__Bacteria;p__Firmicutes;c__Clostridia;o__Clostridiales;f__Ruminococcaceae;g__Anaerotruncus	0.0%	0.0%	0.0%	0.0%	0.0%	0.0%	0.0%	0.0%	0.0%	0.0%	0.0%	0.0%	0.0%
	k__Bacteria;p__Firmicutes;c__Clostridia;o__Clostridiales;f__Ruminococcaceae;g__Cellulosibacter	0.0%	0.0%	0.0%	0.0%	0.0%	0.0%	0.0%	0.0%	0.0%	0.0%	0.0%	0.0%	0.0%
	k__Bacteria;p__Firmicutes;c__Clostridia;o__Clostridiales;f__Ruminococcaceae;g__Oscillospira	0.0%	0.0%	0.0%	0.0%	0.0%	0.0%	0.0%	0.0%	0.0%	0.0%	0.0%	0.0%	0.0%
	k__Bacteria;p__Firmicutes;c__Clostridia;o__Clostridiales;f__Ruminococcaceae;g__Ruminococcus	0.3%	0.9%	0.9%	0.3%	0.5%	0.2%	0.1%	0.3%	0.4%	0.1%	0.1%	0.1%	0.0%
	k__Bacteria;p__Firmicutes;c__Clostridia;o__Clostridiales;f__Symbiobacteriaceae;g__Symbiobacterium	0.0%	0.0%	0.0%	0.0%	0.0%	0.0%	0.0%	0.0%	0.0%	0.0%	0.0%	0.0%	0.0%
	k__Bacteria;p__Firmicutes;c__Clostridia;o__Clostridiales;f__Syntrophomonadaceae;g__Syntrophomonas	2.1%	3.2%	2.6%	2.3%	3.1%	1.8%	1.3%	2.9%	1.9%	1.9%	1.5%	1.3%	1.1%
	k__Bacteria;p__Firmicutes;c__Clostridia;o__Clostridiales;f__Veillonellaceae;g__	0.7%	0.3%	0.3%	0.2%	0.9%	1.2%	0.9%	0.1%	0.7%	0.4%	1.2%	0.9%	0.7%
	k__Bacteria;p__Firmicutes;c__Clostridia;o__Clostridiales;f__Veillonellaceae;g__BSV43	0.0%	0.0%	0.0%	0.0%	0.0%	0.0%	0.0%	0.0%	0.0%	0.0%	0.0%	0.0%	0.0%
	k__Bacteria;p__Firmicutes;c__Clostridia;o__Clostridiales;f__Veillonellaceae;g__Megasphaera	0.0%	0.0%	0.0%	0.0%	0.0%	0.0%	0.0%	0.0%	0.0%	0.0%	0.0%	0.0%	0.0%
	k__Bacteria;p__Firmicutes;c__Clostridia;o__Clostridiales;f__Veillonellaceae;g__Selenomonas	0.0%	0.0%	0.0%	0.0%	0.0%	0.0%	0.0%	0.0%	0.0%	0.0%	0.0%	0.0%	0.0%
	k__Bacteria;p__Firmicutes;c__Clostridia;o__Clostridiales;f__Veillonellaceae;g__Sporomusa	0.0%	0.0%	0.0%	0.0%	0.0%	0.0%	0.0%	0.0%	0.0%	0.0%	0.0%	0.0%	0.0%
	k__Bacteria;p__Firmicutes;c__Clostridia;o__Clostridiales;f__[Acidaminobacteraceae];g__Guggenheimella	0.0%	0.1%	0.0%	0.0%	0.0%	0.0%	0.0%	0.0%	0.1%	0.0%	0.0%	0.0%	0.0%
	k__Bacteria;p__Firmicutes;c__Clostridia;o__Clostridiales;f__[Mogibacteriaceae];Other	0.0%	0.0%	0.0%	0.0%	0.0%	0.0%	0.0%	0.0%	0.0%	0.0%	0.0%	0.0%	0.0%
	k__Bacteria;p__Firmicutes;c__Clostridia;o__Clostridiales;f__[Mogibacteriaceae];g__	0.0%	0.0%	0.0%	0.0%	0.0%	0.1%	0.0%	0.0%	0.0%	0.0%	0.0%	0.0%	0.0%
	k__Bacteria;p__Firmicutes;c__Clostridia;o__Clostridiales;f__[Mogibacteriaceae];g__Anaerovorax	0.0%	0.0%	0.0%	0.0%	0.0%	0.0%	0.0%	0.0%	0.0%	0.0%	0.0%	0.0%	0.0%
	k__Bacteria;p__Firmicutes;c__Clostridia;o__Clostridiales;f__[Tissierellaceae];Other	0.1%	0.1%	0.1%	0.0%	0.2%	0.1%	0.1%	0.0%	0.1%	0.0%	0.1%	0.1%	0.1%
	k__Bacteria;p__Firmicutes;c__Clostridia;o__Clostridiales;f__[Tissierellaceae];g__	0.9%	1.0%	1.0%	0.9%	1.6%	1.2%	0.9%	1.0%	0.8%	0.6%	0.4%	0.6%	0.6%
	k__Bacteria;p__Firmicutes;c__Clostridia;o__Clostridiales;f__[Tissierellaceae];g__GW-34	0.0%	0.0%	0.0%	0.0%	0.0%	0.0%	0.0%	0.0%	0.0%	0.0%	0.0%	0.0%	0.0%
	k__Bacteria;p__Firmicutes;c__Clostridia;o__Clostridiales;f__[Tissierellaceae];g__Gallicola	0.0%	0.0%	0.0%	0.0%	0.0%	0.0%	0.0%	0.0%	0.0%	0.0%	0.0%	0.0%	0.0%
	k__Bacteria;p__Firmicutes;c__Clostridia;o__Clostridiales;f__[Tissierellaceae];g__Sedimentibacter	3.4%	4.4%	3.1%	2.7%	4.9%	3.3%	3.0%	6.8%	1.9%	4.5%	1.7%	1.3%	2.8%
	k__Bacteria;p__Firmicutes;c__Clostridia;o__Clostridiales;f__[Tissierellaceae];g__Sporanaerobacter	0.0%	0.0%	0.0%	0.0%	0.0%	0.0%	0.0%	0.0%	0.0%	0.0%	0.0%	0.0%	0.0%
	k__Bacteria;p__Firmicutes;c__Clostridia;o__Clostridiales;f__[Tissierellaceae];g__Tepidimicrobium	0.0%	0.0%	0.0%	0.0%	0.0%	0.0%	0.0%	0.0%	0.0%	0.0%	0.0%	0.0%	0.0%
	k__Bacteria;p__Firmicutes;c__Clostridia;o__Clostridiales;f__[Tissierellaceae];g__Tissierella_Soehngenia	0.1%	0.1%	0.1%	0.1%	0.1%	0.1%	0.1%	0.1%	0.1%	0.0%	0.0%	0.0%	0.0%
	k__Bacteria;p__Firmicutes;c__Clostridia;o__Halanaerobiales;f__Halanaerobiaceae;g__	0.0%	0.0%	0.0%	0.0%	0.0%	0.0%	0.1%	0.0%	0.0%	0.0%	0.0%	0.0%	0.0%
	k__Bacteria;p__Firmicutes;c__Clostridia;o__MBA08;f__;g__	30.2%	15.5%	22.6%	24.8%	17.9%	37.1%	39.3%	17.3%	28.3%	35.9%	36.9%	38.9%	47.3%
	k__Bacteria;p__Firmicutes;c__Clostridia;o__Natranaerobiales;f__;g__	0.0%	0.0%	0.0%	0.0%	0.0%	0.0%	0.0%	0.0%	0.0%	0.0%	0.0%	0.0%	0.0%
	k__Bacteria;p__Firmicutes;c__Clostridia;o__Natranaerobiales;f__Anaerobrancaceae;Other	0.0%	0.0%	0.0%	0.0%	0.0%	0.0%	0.0%	0.0%	0.0%	0.0%	0.0%	0.0%	0.0%
	k__Bacteria;p__Firmicutes;c__Clostridia;o__Natranaerobiales;f__Anaerobrancaceae;g__	0.2%	0.1%	0.2%	0.2%	0.1%	0.2%	0.1%	0.1%	0.2%	0.3%	0.1%	0.2%	0.2%
	k__Bacteria;p__Firmicutes;c__Clostridia;o__Natranaerobiales;f__Anaerobrancaceae;g__A55_D21	0.0%	0.0%	0.0%	0.0%	0.0%	0.0%	0.0%	0.0%	0.0%	0.0%	0.0%	0.0%	0.0%
	k__Bacteria;p__Firmicutes;c__Clostridia;o__Natranaerobiales;f__Anaerobrancaceae;g__Dethiobacter	0.2%	0.2%	0.1%	0.2%	0.1%	0.1%	0.1%	0.2%	0.2%	0.1%	0.2%	0.2%	0.2%
	k__Bacteria;p__Firmicutes;c__Clostridia;o__Natranaerobiales;f__Contubernalisaceae;Other	0.0%	0.0%	0.0%	0.0%	0.0%	0.0%	0.0%	0.0%	0.0%	0.0%	0.0%	0.0%	0.0%
	k__Bacteria;p__Firmicutes;c__Clostridia;o__Natranaerobiales;f__ML1228J-1;g__	0.3%	0.1%	0.2%	0.3%	0.2%	0.2%	0.3%	0.3%	0.1%	0.2%	0.3%	0.6%	0.3%
	k__Bacteria;p__Firmicutes;c__Clostridia;o__Natranaerobiales;f__YAB3B13;g__	0.1%	0.1%	0.1%	0.1%	0.0%	0.0%	0.0%	0.1%	0.1%	0.1%	0.1%	0.1%	0.1%
	k__Bacteria;p__Firmicutes;c__Clostridia;o__OPB54;f__;g__	0.7%	0.5%	0.7%	1.7%	0.2%	0.3%	0.8%	0.6%	1.0%	0.7%	0.4%	0.6%	0.5%
	k__Bacteria;p__Firmicutes;c__Clostridia;o__SHA-98;Other;Other	0.0%	0.0%	0.0%	0.0%	0.0%	0.0%	0.0%	0.0%	0.0%	0.0%	0.0%	0.0%	0.0%
	k__Bacteria;p__Firmicutes;c__Clostridia;o__SHA-98;f__;g__	7.1%	1.2%	2.8%	3.1%	6.8%	9.3%	10.2%	1.5%	4.8%	5.1%	11.8%	14.9%	13.7%
	k__Bacteria;p__Firmicutes;c__Clostridia;o__SHA-98;f__D2;g__	0.8%	0.3%	0.6%	0.6%	0.5%	1.3%	0.9%	0.3%	0.3%	0.4%	1.1%	1.8%	1.2%
	k__Bacteria;p__Firmicutes;c__Clostridia;o__Thermoanaerobacterales;f__;g__	0.7%	0.6%	0.7%	0.9%	0.4%	0.7%	0.6%	0.5%	0.8%	0.6%	0.6%	0.7%	0.9%
	k__Bacteria;p__Firmicutes;c__Clostridia;o__Thermoanaerobacterales;f__Thermoanaerobacteraceae;g__	0.3%	0.4%	0.5%	0.4%	0.3%	0.3%	0.3%	0.4%	0.4%	0.4%	0.2%	0.3%	0.3%
	k__Bacteria;p__Firmicutes;c__Clostridia;o__Thermoanaerobacterales;f__Thermoanaerobacteraceae;g__Moorella	0.0%	0.0%	0.0%	0.0%	0.0%	0.0%	0.0%	0.0%	0.0%	0.0%	0.0%	0.0%	0.0%
	k__Bacteria;p__Firmicutes;c__Clostridia;o__Thermoanaerobacterales;f__Thermoanaerobacteraceae;g__Thermacetogenium	0.1%	0.0%	0.0%	0.0%	0.1%	0.1%	0.1%	0.0%	0.0%	0.0%	0.1%	0.1%	0.1%
	k__Bacteria;p__Firmicutes;c__Erysipelotrichi;o__Erysipelotrichales;f__Erysipelotrichaceae;g__	0.1%	0.0%	0.1%	0.1%	0.0%	0.0%	0.0%	0.1%	0.1%	0.1%	0.0%	0.0%	0.0%
	k__Bacteria;p__Firmicutes;c__Erysipelotrichi;o__Erysipelotrichales;f__Erysipelotrichaceae;g__L7A_E11	0.0%	0.0%	0.0%	0.0%	0.0%	0.0%	0.0%	0.0%	0.0%	0.0%	0.0%	0.0%	0.0%
	k__Bacteria;p__Firmicutes;c__Erysipelotrichi;o__Erysipelotrichales;f__Erysipelotrichaceae;g__RFN20	0.8%	0.6%	1.0%	0.7%	0.8%	1.1%	1.2%	0.9%	1.1%	0.9%	0.3%	0.4%	0.4%
	k__Bacteria;p__Firmicutes;c__OPB54;o__;f__;g__	1.7%	1.5%	2.4%	2.0%	0.8%	1.6%	1.3%	1.3%	1.6%	3.1%	1.8%	1.9%	1.4%
	k__Bacteria;p__Gemmatimonadetes;c__Gemm-5;o__;f__;g__	0.0%	0.0%	0.0%	0.0%	0.0%	0.0%	0.0%	0.0%	0.0%	0.0%	0.0%	0.0%	0.0%
	k__Bacteria;p__Gemmatimonadetes;c__Gemmatimonadetes;o__;f__;g__	0.0%	0.0%	0.0%	0.0%	0.0%	0.0%	0.0%	0.0%	0.0%	0.0%	0.0%	0.0%	0.0%

k__Bacteria;p__Lentisphaerae;c__[Lentisphaeria];o__Z20;f__R4-45B;g__	0.0%	0.0%	0.0%	0.0%	0.0%	0.0%	0.0%	0.0%	0.0%	0.0%	0.0%	0.0%	0.0%
k__Bacteria;p__NKB19;c__noFP_H4;o__;f__;g__	0.0%	0.0%	0.0%	0.0%	0.0%	0.0%	0.0%	0.0%	0.0%	0.0%	0.0%	0.0%	0.0%
k__Bacteria;p__OD1;c__;o__;f__;g__	0.0%	0.0%	0.0%	0.0%	0.0%	0.0%	0.0%	0.0%	0.0%	0.0%	0.0%	0.0%	0.0%
k__Bacteria;p__OD1;c__ABY1;o__;f__;g__	0.0%	0.1%	0.0%	0.0%	0.0%	0.0%	0.0%	0.0%	0.0%	0.0%	0.0%	0.0%	0.0%
k__Bacteria;p__OP11;c__WCHB1-64;o__K2-4-19;f__;g__	0.0%	0.0%	0.0%	0.0%	0.0%	0.0%	0.0%	0.0%	0.0%	0.0%	0.0%	0.0%	0.0%
k__Bacteria;p__OP11;c__WCHB1-64;o__d153;f__;g__	0.0%	0.0%	0.0%	0.0%	0.0%	0.0%	0.0%	0.0%	0.0%	0.0%	0.0%	0.0%	0.0%
k__Bacteria;p__OP9;c__JS1;o__BA021;f__;g__	0.0%	0.0%	0.0%	0.0%	0.0%	0.0%	0.0%	0.0%	0.0%	0.0%	0.0%	0.0%	0.0%
k__Bacteria;p__OP9;c__OPB46;o__OPB72;f__TIBD11;g__	0.0%	0.0%	0.0%	0.1%	0.0%	0.0%	0.1%	0.0%	0.0%	0.0%	0.1%	0.0%	0.1%
k__Bacteria;p__OP9;c__OPB46;o__SHA-1;f__;g__	0.1%	0.1%	0.1%	0.2%	0.0%	0.0%	0.0%	0.1%	0.1%	0.1%	0.0%	0.0%	0.0%
k__Bacteria;p__Planctomycetes;c__Planctomycetia;o__Pirellulales;f__Pirellulaceae;g__	0.0%	0.0%	0.0%	0.0%	0.0%	0.0%	0.0%	0.0%	0.0%	0.0%	0.0%	0.0%	0.0%
k__Bacteria;p__Proteobacteria;c__Alphaproteobacteria;o__;f__;g__	0.0%	0.0%	0.0%	0.0%	0.0%	0.0%	0.0%	0.0%	0.0%	0.0%	0.0%	0.0%	0.0%
k__Bacteria;p__Proteobacteria;c__Alphaproteobacteria;o__Caulobacterales;f__Caulobacteraceae;g__Mycoplana	0.0%	0.0%	0.0%	0.0%	0.0%	0.0%	0.0%	0.0%	0.0%	0.0%	0.0%	0.0%	0.0%
k__Bacteria;p__Proteobacteria;c__Alphaproteobacteria;o__Rhizobiales;f__Hyphomicrobiaceae;g__	0.0%	0.0%	0.0%	0.0%	0.0%	0.0%	0.0%	0.0%	0.0%	0.0%	0.0%	0.0%	0.0%
k__Bacteria;p__Proteobacteria;c__Alphaproteobacteria;o__Rhizobiales;f__Hyphomicrobiaceae;g__Hyphomicrobium	0.0%	0.0%	0.0%	0.0%	0.0%	0.0%	0.0%	0.0%	0.0%	0.0%	0.0%	0.0%	0.0%
k__Bacteria;p__Proteobacteria;c__Alphaproteobacteria;o__Rhizobiales;f__Phyllobacteriaceae;Other	0.0%	0.0%	0.0%	0.0%	0.0%	0.0%	0.0%	0.0%	0.0%	0.0%	0.0%	0.0%	0.0%
k__Bacteria;p__Proteobacteria;c__Alphaproteobacteria;o__Rhodobacterales;f__Hyphomonadaceae;g__	0.0%	0.0%	0.0%	0.0%	0.0%	0.0%	0.0%	0.0%	0.0%	0.0%	0.0%	0.0%	0.0%
k__Bacteria;p__Proteobacteria;c__Alphaproteobacteria;o__Rhodobacterales;f__Rhodobacteraceae;Other	0.0%	0.0%	0.0%	0.0%	0.0%	0.0%	0.0%	0.0%	0.0%	0.0%	0.0%	0.0%	0.0%
k__Bacteria;p__Proteobacteria;c__Alphaproteobacteria;o__Rhodospirillales;f__Acetobacteraceae;Other	0.0%	0.0%	0.0%	0.0%	0.0%	0.0%	0.0%	0.0%	0.0%	0.0%	0.0%	0.0%	0.0%
k__Bacteria;p__Proteobacteria;c__Alphaproteobacteria;o__Rhodospirillales;f__Rhodospirillaceae;g__Azospirillum	0.0%	0.0%	0.0%	0.0%	0.0%	0.0%	0.0%	0.0%	0.0%	0.0%	0.0%	0.0%	0.0%
k__Bacteria;p__Proteobacteria;c__Alphaproteobacteria;o__Rickettsiales;f__;g__	0.0%	0.0%	0.0%	0.0%	0.0%	0.0%	0.0%	0.0%	0.0%	0.0%	0.0%	0.0%	0.0%
k__Bacteria;p__Proteobacteria;c__Betaproteobacteria;o__Burkholderiales;f__Alcaligenaceae;g__	0.0%	0.0%	0.0%	0.0%	0.0%	0.0%	0.0%	0.0%	0.0%	0.0%	0.0%	0.0%	0.0%
k__Bacteria;p__Proteobacteria;c__Betaproteobacteria;o__Burkholderiales;f__Comamonadaceae;g__	0.0%	0.0%	0.0%	0.0%	0.0%	0.0%	0.0%	0.0%	0.0%	0.0%	0.0%	0.0%	0.0%
k__Bacteria;p__Proteobacteria;c__Betaproteobacteria;o__Burkholderiales;f__Comamonadaceae;g__Comamonas	0.0%	0.0%	0.0%	0.0%	0.0%	0.0%	0.0%	0.0%	0.0%	0.0%	0.0%	0.0%	0.0%
k__Bacteria;p__Proteobacteria;c__Betaproteobacteria;o__Burkholderiales;f__Comamonadaceae;g__Lampropedia	0.0%	0.0%	0.0%	0.0%	0.0%	0.0%	0.0%	0.0%	0.0%	0.0%	0.0%	0.0%	0.0%
k__Bacteria;p__Proteobacteria;c__Deltaproteobacteria;o__Desulfuromonadales;f__Geobacteraceae;g__Geobacter	0.0%	0.0%	0.0%	0.0%	0.0%	0.0%	0.0%	0.0%	0.0%	0.0%	0.0%	0.0%	0.0%
k__Bacteria;p__Proteobacteria;c__Deltaproteobacteria;o__Desulfuromonadales;f__Pelobacteraceae;g__	0.0%	0.0%	0.0%	0.0%	0.0%	0.0%	0.0%	0.0%	0.0%	0.0%	0.0%	0.0%	0.0%
k__Bacteria;p__Proteobacteria;c__Deltaproteobacteria;o__GW-28;f__;g__	0.0%	0.0%	0.0%	0.0%	0.0%	0.0%	0.0%	0.0%	0.0%	0.0%	0.0%	0.0%	0.0%
k__Bacteria;p__Proteobacteria;c__Deltaproteobacteria;o__Myxococcales;f__;g__	0.0%	0.0%	0.0%	0.1%	0.0%	0.0%	0.0%	0.0%	0.0%	0.0%	0.0%	0.0%	0.0%
k__Bacteria;p__Proteobacteria;c__Deltaproteobacteria;o__Myxococcales;f__Polyangiaceae;g__Chondromyces	0.0%	0.0%	0.0%	0.0%	0.0%	0.0%	0.0%	0.0%	0.0%	0.0%	0.0%	0.0%	0.0%
k__Bacteria;p__Proteobacteria;c__Deltaproteobacteria;o__Syntrophobacterales;f__Syntrophaceae;g__	0.0%	0.0%	0.0%	0.0%	0.0%	0.0%	0.0%	0.0%	0.0%	0.0%	0.0%	0.0%	0.0%
k__Bacteria;p__Proteobacteria;c__Gammaproteobacteria;o__Aeromonadales;f__Succinivibrionaceae;g__Succinivibrio	0.0%	0.0%	0.0%	0.0%	0.0%	0.0%	0.0%	0.0%	0.0%	0.0%	0.0%	0.0%	0.0%
k__Bacteria;p__Proteobacteria;c__Gammaproteobacteria;o__Enterobacteriales;f__Enterobacteriaceae;Other	0.0%	0.0%	0.0%	0.0%	0.0%	0.0%	0.0%	0.0%	0.0%	0.0%	0.0%	0.0%	0.0%
k__Bacteria;p__Proteobacteria;c__Gammaproteobacteria;o__Enterobacteriales;f__Enterobacteriaceae;g__Erwinia	0.0%	0.0%	0.0%	0.0%	0.0%	0.0%	0.0%	0.0%	0.0%	0.0%	0.0%	0.0%	0.0%
k__Bacteria;p__Proteobacteria;c__Gammaproteobacteria;o__Enterobacteriales;f__Enterobacteriaceae;g__Klebsiella	0.0%	0.0%	0.0%	0.0%	0.0%	0.0%	0.0%	0.0%	0.0%	0.0%	0.0%	0.0%	0.0%
k__Bacteria;p__Proteobacteria;c__Gammaproteobacteria;o__Methylococcales;f__Methylococcaceae;g__Methylocaldum	0.0%	0.0%	0.0%	0.0%	0.0%	0.0%	0.0%	0.0%	0.0%	0.0%	0.0%	0.0%	0.0%
k__Bacteria;p__Proteobacteria;c__Gammaproteobacteria;o__Pseudomonadales;f__Pseudomonadaceae;g__Pseudomonas	0.0%	0.0%	0.0%	0.0%	0.0%	0.0%	0.0%	0.0%	0.0%	0.0%	0.0%	0.0%	0.0%
k__Bacteria;p__Proteobacteria;c__Gammaproteobacteria;o__Xanthomonadales;f__Xanthomonadaceae;Other	0.0%	0.0%	0.0%	0.0%	0.0%	0.0%	0.0%	0.0%	0.0%	0.0%	0.0%	0.0%	0.0%
k__Bacteria;p__Proteobacteria;c__Gammaproteobacteria;o__Xanthomonadales;f__Xanthomonadaceae;g__	0.0%	0.0%	0.0%	0.0%	0.0%	0.0%	0.0%	0.0%	0.0%	0.0%	0.0%	0.0%	0.0%
k__Bacteria;p__Proteobacteria;c__Gammaproteobacteria;o__Xanthomonadales;f__Xanthomonadaceae;g__Luteimonas	0.0%	0.0%	0.0%	0.0%	0.0%	0.0%	0.0%	0.0%	0.0%	0.0%	0.0%	0.0%	0.0%
k__Bacteria;p__Proteobacteria;c__Gammaproteobacteria;o__Xanthomonadales;f__Xanthomonadaceae;g__Lysobacter	0.0%	0.0%	0.0%	0.0%	0.0%	0.0%	0.0%	0.0%	0.0%	0.0%	0.0%	0.0%	0.0%
k__Bacteria;p__SR1;c__;o__;f__;g__	0.4%	0.4%	0.1%	0.1%	0.2%	1.4%	1.1%	0.1%	0.4%	0.1%	0.1%	0.1%	0.3%
k__Bacteria;p__Spirochaetes;c__MVP-15;o__PL-11B10;f__;g__	0.0%	0.0%	0.0%	0.0%	0.0%	0.0%	0.0%	0.0%	0.0%	0.0%	0.0%	0.0%	0.0%
k__Bacteria;p__Spirochaetes;c__Spirochaetes;o__Sphaerochaetales;f__Sphaerochaetaceae;g__	0.0%	0.0%	0.0%	0.0%	0.0%	0.0%	0.0%	0.0%	0.0%	0.0%	0.0%	0.0%	0.0%
k__Bacteria;p__Spirochaetes;c__Spirochaetes;o__Sphaerochaetales;f__Sphaerochaetaceae;g__Sphaerochaeta	0.0%	0.0%	0.0%	0.1%	0.0%	0.0%	0.0%	0.1%	0.0%	0.0%	0.0%	0.0%	0.0%
k__Bacteria;p__Spirochaetes;c__Spirochaetes;o__Spirochaetales;f__Spirochaetaceae;g__	0.0%	0.0%	0.0%	0.0%	0.0%	0.0%	0.0%	0.0%	0.0%	0.0%	0.0%	0.0%	0.0%
k__Bacteria;p__Spirochaetes;c__Spirochaetes;o__Spirochaetales;f__Spirochaetaceae;g__HAW-RM37	0.0%	0.0%	0.0%	0.0%	0.0%	0.0%	0.0%	0.0%	0.0%	0.0%	0.0%	0.0%	0.0%
k__Bacteria;p__Spirochaetes;c__Spirochaetes;o__Spirochaetales;f__Spirochaetaceae;g__Treponema	0.2%	0.2%	0.2%	0.5%	0.4%	0.5%	0.2%	0.1%	0.5%	0.1%	0.1%	0.1%	0.0%
k__Bacteria;p__Synergistetes;c__Synergistia;o__Synergistales;Other;Other	0.0%	0.0%	0.0%	0.0%	0.0%	0.0%	0.0%	0.0%	0.0%	0.0%	0.0%	0.0%	0.0%
k__Bacteria;p__Synergistetes;c__Synergistia;o__Synergistales;f__Anaerobaculaceae;g__Anaerobaculum	0.3%	0.1%	0.1%	0.1%	0.3%	0.4%	0.3%	0.1%	0.1%	0.3%	0.5%	0.5%	0.5%
k__Bacteria;p__Synergistetes;c__Synergistia;o__Synergistales;f__Dethiosulfovibrionaceae;g__	0.0%	0.0%	0.0%	0.0%	0.0%	0.0%	0.0%	0.0%	0.0%	0.0%	0.0%	0.0%	0.0%
k__Bacteria;p__Synergistetes;c__Synergistia;o__Synergistales;f__Dethiosulfovibrionaceae;g__Aminobacterium	0.0%	0.0%	0.0%	0.0%	0.0%	0.0%	0.0%	0.0%	0.0%	0.0%	0.0%	0.0%	0.0%
k__Bacteria;p__Synergistetes;c__Synergistia;o__Synergistales;f__Dethiosulfovibrionaceae;g__HA73	0.0%	0.0%	0.0%	0.0%	0.0%	0.0%	0.0%	0.0%	0.0%	0.0%	0.0%	0.0%	0.0%
k__Bacteria;p__Synergistetes;c__Synergistia;o__Synergistales;f__Synergistaceae;g__vadinCA02	0.2%	0.3%	0.3%	0.5%	0.1%	0.1%	0.1%	0.5%	0.2%	0.3%	0.1%	0.1%	0.1%
k__Bacteria;p__Tenericutes;c__Mollicutes;Other;Other;Other	0.0%	0.0%	0.0%	0.0%	0.0%	0.0%	0.0%	0.0%	0.0%	0.0%	0.0%	0.0%	0.0%
k__Bacteria;p__Tenericutes;c__Mollicutes;o__;f__;g__	0.0%	0.0%	0.0%	0.0%	0.0%	0.0%	0.0%	0.0%	0.0%	0.0%	0.0%	0.0%	0.0%
k__Bacteria;p__Tenericutes;c__Mollicutes;o__Acholeplasmatales;f__Acholeplasmataceae;g__	0.1%	0.1%	0.1%	0.1%	0.2%	0.2%	0.1%	0.1%	0.1%	0.1%	0.1%	0.1%	0.1%
k__Bacteria;p__Tenericutes;c__Mollicutes;o__Acholeplasmatales;f__Acholeplasmataceae;g__Acholeplasma	0.6%	1.1%	1.4%	0.6%	0.5%	0.5%	0.6%	0.7%	0.5%	0.5%	0.2%	0.1%	0.2%
k__Bacteria;p__Tenericutes;c__Mollicutes;o__RF39;f__;g__	0.0%	0.0%	0.0%	0.0%	0.0%	0.0%	0.0%	0.0%	0.0%	0.0%	0.0%	0.0%	0.0%
k__Bacteria;p__Tenericutes;c__RF3;o__ML615J-28;f__;g__	1.5%	0.9%	1.5%	1.8%	2.0%	1.4%	1.0%	1.3%	1.7%	2.0%	1.8%	1.5%	0.9%
k__Bacteria;p__Thermotogae;c__Thermotogae;o__Thermotogales;f__Thermotogaceae;g__Kosmotoga	0.0%	0.0%	0.0%	0.0%	0.0%	0.0%	0.0%	0.0%	0.0%	0.0%	0.0%	0.0%	0.0%
k__Bacteria;p__Thermotogae;c__Thermotogae;o__Thermotogales;f__Thermotogaceae;g__S1	0.0%	0.0%	0.0%	0.0%	0.0%	0.0%	0.0%	0.0%	0.0%	0.0%	0.0%	0.0%	0.0%
k__Bacteria;p__Verrucomicrobia;c__Opitutae;o__[Cerasicoccales];f__[Cerasicoccaceae];g__	0.0%	0.0%	0.0%	0.0%	0.0%	0.0%	0.0%	0.0%	0.0%	0.0%	0.0%	0.0%	0.0%
k__Bacteria;p__Verrucomicrobia;c__Verruco-5;o__;f__;g__	0.0%	0.0%	0.0%	0.0%	0.0%	0.0%	0.0%	0.0%	0.0%	0.0%	0.0%	0.0%	0.0%
k__Bacteria;p__Verrucomicrobia;c__Verruco-5;o__LD1-PB3;f__;g__	0.0%	0.0%	0.0%	0.0%	0.0%	0.0%	0.0%	0.0%	0.0%	0.0%	0.0%	0.0%	0.0%
k__Bacteria;p__Verrucomicrobia;c__Verruco-5;o__WCHB1-41;f__;g__	0.0%	0.0%	0.0%	0.0%	0.0%	0.0%	0.0%	0.0%	0.0%	0.0%	0.0%	0.0%	0.0%
k__Bacteria;p__Verrucomicrobia;c__Verruco-5;o__WCHB1-41;f__RFP12;g__	0.0%	0.0%	0.0%	0.0%	0.0%	0.0%	0.0%	0.0%	0.0%	0.0%	0.0%	0.0%	0.0%
k__Bacteria;p__Verrucomicrobia;c__[Pedosphaerae];o__[Pedosphaerales];f__;g__	0.0%	0.0%	0.0%	0.0%	0.0%	0.0%	0.0%	0.0%	0.0%	0.0%	0.0%	0.0%	0.0%
k__Bacteria;p__Verrucomicrobia;c__[Pedosphaerae];o__[Pedosphaerales];f__R4-41B;g__	0.0%	0.0%	0.0%	0.0%	0.0%	0.0%	0.0%	0.0%	0.0%	0.0%	0.0%	0.0%	0.0%
k__Bacteria;p__WPS-2;c__;o__;f__;g__	0.0%	0.0%	0.0%	0.0%	0.0%	0.0%	0.0%	0.0%	0.1%	0.1%	0.0%	0.0%	0.0%
k__Bacteria;p__WS6;c__SC72;o__A-2AF;f__;g__	0.0%	0.0%	0.0%	0.0%	0.0%	0.0%	0.0%	0.0%	0.0%	0.0%	0.0%	0.0%	0.0%
k__Bacteria;p__WS6;c__SC72;o__WCHB1-15;f__;g__	0.0%	0.0%	0.0%	0.0%	0.0%	0.0%	0.0%	0.0%	0.0%	0.0%	0.0%	0.0%	0.0%
k__Bacteria;p__WWE1;c__[Cloacamonae];o__[Cloacamonales];f__CW-1;g__	0.0%	0.0%	0.0%	0.0%	0.0%	0.0%	0.0%	0.0%	0.0%	0.0%	0.0%	0.0%	0.0%
k__Bacteria;p__WWE1;c__[Cloacamonae];o__[Cloacamonales];f__[Cloacamonaceae];Other	0.2%	0.0%	0.1%	0.0%	0.0%	0.0%	1.6%	0.0%	0.2%	0.1%	0.0%	0.0%	0.3%
k__Bacteria;p__WWE1;c__[Cloacamonae];o__[Cloacamonales];f__[Cloacamonaceae];g__BHB21	2.6%	3.6%	4.3%	5.3%	0.2%	0.1%	0.1%	5.3%	5.4%	6.5%	0.1%	0.1%	0.0%
k__Bacteria;p__WWE1;c__[Cloacamonae];o__[Cloacamonales];f__[Cloacamonaceae];g__Candidatus Cloacamonas	0.0%	0.0%	0.0%	0.0%	0.0%	0.0%	0.0%	0.0%	0.0%	0.0%	0.0%	0.0%	0.0%
k__Bacteria;p__WWE1;c__[Cloacamonae];o__[Cloacamonales];f__[Cloacamonaceae];g__W22	0.0%	0.0%	0.0%	0.0%	0.0%	0.0%	0.0%	0.0%	0.0%	0.0%	0.0%	0.0%	0.0%
k__Bacteria;p__WWE1;c__[Cloacamonae];o__[Cloacamonales];f__[Cloacamonaceae];g__W5	0.0%	0.0%	0.0%	0.0%	0.1%	0.0%	0.0%	0.1%	0.0%	0.0%	0.0%	0.0%	0.0%
k__Bacteria;p__[Thermi];c__Deinococci;o__Deinococcales;f__Trueperaceae;g__B-42	0.0%	0.0%	0.0%	0.0%	0.0%	0.0%	0.0%	0.0%	0.0%	0.0%	0.0%	0.0%	0.0%
